# Phenotype switching in highly invasive resistant to vemurafenib and cobimetinib melanoma cells

**DOI:** 10.1186/s12964-025-02452-0

**Published:** 2025-10-21

**Authors:** Aleksandra Simiczyjew, Magdalena Kot, Michał Majkowski, Marcin Ziętek, Rafał Matkowski, Dorota Nowak

**Affiliations:** 1 Department of Cell Pathology, Faculty of Biotechnology, University of Wroclaw, Joliot-Curie 14a, Wroclaw, 50-383 Poland; 2 Department of Oncology, Division of Surgical Oncology, Wroclaw Medical University, Plac Hirszfelda 12, Wroclaw, 53-413 Poland; 3 Lower Silesian Oncology, Pulmonology, and Hematology Center, Plac Hirszfelda 12, Wroclaw, 53-413 Poland

**Keywords:** Vemurafenib, Cobimetinib, BRAF inhibitor, MEK inhibitor, Drug resistance, Melanoma

## Abstract

**Background:**

A mutation in the BRAF (serine/threonine-protein kinase B-raf) gene is most often responsible for the progression of melanoma. A breakthrough in its treatment was the application of BRAF and MEK (mitogen-activated protein kinase kinase) inhibitors. Unfortunately, the effectiveness of this therapy is limited due to rapidly emerging resistance to the drugs. We derived two melanoma cell lines resistant to vemurafenib (a BRAF inhibitor)/cobimetinib (an MEK inhibitor). Due to the significant impact of invasion on cancer progression, we focused our further research on this process.

**Methods:**

Cell migration and invasion were assessed via the scratch wound assay. Selected proteins’ level as well as the activation of focal adhesion kinase (FAK) were evaluated using Western blotting. The expression of the selected genes was examined by qRT-PCR. The focal adhesions parameters, actin polymerization ratio, as well as YAP/TAZ (Yes-associated protein/transcriptional co-activator with PDZ-binding motif), invadopodia, and β and γ actin localization were analyzed using confocal microscopy. The composition and activity of proteases secreted by cells were determined using a human protease array and gelatin zymography. In addition, cell adhesion and matrix metalloproteinase (MMP14) activity were assessed using appropriate assays.

**Results:**

Our analysis showed a greater capacity for migration and invasion of resistant melanoma cells compared to controls, as well as an increase in the level of RUNX2 (runt-related transcription factor 2). Moreover, examined cells exhibited higher adhesion to the surface and were more spread. These cells also formed more focal adhesions. Furthermore, we noticed an increased level of α-parvin and vinculin in resistant cells, as well as an elevated activation of FAK (focal adhesion kinase). Resistance was additionally accompanied by rearrangement of the actin cytoskeleton. Examined cells formed more stress fibers compared to control cells. YAP/TAZ localization became much more nuclear in the resistant ones. The amount of invadopodia was increased, which was reflected by elevated secretion and activation of proteases, as well as altered expression of their inhibitors.

**Conclusions:**

In summary, our study characterized a significantly more invasive phenotype of double-resistant cell lines compared to melanoma cells sensitive to BRAF and MEK inhibitors. Successful inhibition of this phenotype could result in more effective therapy and thus a better prognosis for patients.

**Supplementary Information:**

The online version contains supplementary material available at 10.1186/s12964-025-02452-0.

## Introduction

 The high aggressiveness of melanoma is mainly caused by the presence of a mutated form of BRAF (serine/threonine-protein kinase B-raf) kinase in approximately 50% of patients [1]. This mutation, which results in a valine to glutamic acid substitution at position 600 of the polypeptide chain (V600E), makes the aforementioned kinase 500 times more active. It leads to a significantly elevated activation of the MAPK (mitogen-activated protein kinase) signaling pathway, which results in increased proliferation and migration of cancer cells, and therefore stimulates the progression of melanoma [2, 3]. In addition to BRAF kinase, another important element of the MAPK pathway, which is frequently overactivated in melanoma cells, is MEK (mitogen-activated protein kinase kinase). A combination of BRAF/MEK inhibitors is one of the most commonly used methods in treating advanced melanoma [4]. Unfortunately, the rapidly emerging resistance to treatment among patients limits the effectiveness of applied therapy. It is known that this phenomenon may result from, among others, reactivation of the MAPK pathway, amplification of BRAF V600E, MEK, mutations of NRAS (N-ras proto-oncogene), or alternative splicing [5]. However, the mechanisms associated with the resistance of melanoma cells to BRAF/MEK inhibitors have not yet been thoroughly understood. We have obtained and characterized two melanoma cell lines (WM9 and Hs294T) resistant to inhibitors of BRAF (vemurafenib) and MEK (cobimetinib) kinases. This inhibitors’ combination was selected because it is routinely utilized in treating melanoma patients with the BRAF mutation [6]. In the previous studies, we identified the basic characteristics of these cells associated with their resistance to treatment. We found that despite the presence of BRAF/MEK inhibitors, they exhibit elevated survival and activation of ERK. Moreover, derived cells demonstrated raised expression of growth factors, among others, hepatocyte growth factor receptor (MET), epidermal growth factor receptor (EGFR), as well as elevated activation of p38 mitogen-activated protein kinases, c-Jun N-terminal kinase (JNK), and protein kinase B (AKT) signaling pathways. Additionally, resistant cells exhibited raised expression of genes encoding proteins engaged in drug metabolism and transport. Furthermore, in cells resistant to cobimetinib/vemurafenib, we observed features of cancer stem cells and epithelial-mesenchymal transition, decreased proliferation rate, and increased cytokine secretion [7]. Here, we performed a comprehensive characterization of melanoma cell lines resistant to BRAFi/MEKi (BRAF inhibitor/MEK inhibitor) in terms of their adhesive, migrative, and proteolytic abilities, which are essential during cell invasion and thus metastasis. Listed processes that are very common in melanoma and ultimately lead to deaths among patients. For this reason, understanding the mechanisms that drive invasion could help to develop new forms of therapy that would inhibit this phenomenon and thus extend the lifespan of patients.

## Materials and methods

### Acquisition of resistant melanoma cell lines

Resistant cells were obtained from two metastatic melanoma cell lines: WM9 (purchased in 2018 from Rockland Immunochemicals) and Hs294T (purchased in 2019 from ATCC). Both WM9 and Hs294T cells have the BRAF V600E mutation [8, 9]. Cells were cultured in Dulbecco’s Modified Eagle Medium (DMEM; IITD PAN) with the addition of 10% fetal bovine serum (FBS; Thermo Fisher), 2mM glutamine (Thermo Fisher), and antibiotic-antimycotic solution (10.000 U/ml penicillin, 10 mg/ml streptomycin, 25 µg/ml amphotericin B; Thermo Fisher). Cells were cultured in optimal growth conditions (37 °C and 5%CO_2_/95% humidified air). Cells were passaged using 0.25% trypsin/0.05% EDTA (ethylenediaminetetraacetic acid) solution (IITD PAN).

To obtain resistant melanoma cell lines, naïve cells were treated with increasing concentrations of MEK inhibitor - cobimetinib (Selleck Chemicals LLC) and BRAF inhibitor - vemurafenib (Santa Cruz Biotechnology). Our previous work described the exact procedure for developing resistant cells in detail [7]. Control WM9 and Hs294T cell lines were obtained by treating naïve cells with increasing concentrations of the solvent of the inhibitors - dimethyl sulfoxide (DMSO; AppliChem). The acquired resistant cells were grown in a medium containing 0.4µM of both inhibitors, while control cells were cultured in a medium containing 0,01% DMSO. Resistant cell lines were authenticated using the Short Tandem Repeat (STR) profiling method in 2023 by ATCC (American Type Culture Collection). All cell lines were repeatedly verified for mycoplasma contamination.

### 2D and 3D wound healing

Cells were seeded into 1 mg/ml Matrigel-coated 96-well plates (IncuCyte ImageLock, Sartorius) and cultured for 24 h. Next, scratches were made using a Wound Maker™ (Essen Bioscience) simultaneously in all wells. To evaluate the invasion, the cells and the area devoid of cells was covered with another Matrigel layer. Next, to the cells was added the cell culture medium directly (2D migration assay) or on top of 3D Matrigel matrices (3D invasion assay). Phase-contrast images were taken in an IncuCyte^®^ Live-Cell Analysis System every 2 h. Cells were able to cover the scratch for 24 h (2D conditions) or 48 h (3D conditions). Images were analyzed utilizing the IncuCyte^®^ Scratch Wound Cell Migration Software Module (Sartorius), and representative ones are shown in the appropriate Figures. The wound closure represents the rise of the area, which was covered by the cells. Experiments were performed in three biological repetitions. Each condition consisted of three technical replicates.

### Western blotting analysis

Protein extracts from control and resistant WM9 and Hs294T melanoma cells were achieved with urea buffer (50 mM Tris, pH 7.4, 1 mM dichlorodiphenyltrichloroethane, 74 mM urea, 5% sodium dodecyl sulfate (SDS), 8.6% sucrose) with protease and phosphatase inhibitors cocktails (Sigma Aldrich). To measure the protein concentration, the bicinchoninic acid (BCA) procedure (Thermo Fisher) was applied. Samples containing the identical amount of protein (10 µg for cell lysates and 5 µg for conditioned media) were separated using a 10% polyacrylamide gel by SDS-PAGE electrophoresis. Next, proteins were transferred to nitrocellulose membranes.

Primary antibodies directed against RUNX2 (Santa Cruz Biotechnology, sc-390351, 1:200), α parvin (Cell Signaling Technologies, #4026, 1:1000), vinculin (Bio-Rad, MCA465GA, 1:200), FAK (Santa Cruz Biotechnology, sc-932, 1:200), phosphorylated FAK (pFAK; Invitrogen, #44-625G, 1:1000), β actin (Bio-Rad, MCA5775GA, 1:1000), γ actin (Bio-Rad, MCA5776GA, 1:100), matrix metalloproteinase 2 (MMP2; GeneTex, GTX104577, 1:500), matrix metalloproteinase 9 (MMP9; Abcam, ab76003, 1:1000), and secondary antibodies (directed against rabbit and mouse) linked with horseradish peroxidase (Cell Signaling Technologies, 7076 and 7074, 1:4000) were applied as specified by the manufacturers’ instructions. Next, blots were developed with Clarity Max Western ECL Substrate (Bio-Rad) or Clarity Western ECL Substrate (Bio-Rad) and scanned using ChemiDoc (Bio-Rad). Results were evaluated with ImageLab Software (v. 6.0, Bio-Rad) and normalized to the total protein content, which was assessed by Ponceau S staining. At least three separate experiments were conducted, based on three biological replicates. The figures show representative membranes.

### Cytochemical staining

The cytochemical staining and fluorescence were utilized to evaluate the subcellular localization of selected proteins, actin filaments, and cell nuclei. Cells were rinsed with phosphate-buffered saline (PBS), fixed with 4% formaldehyde (Sigma Aldrich), again washed with PBS, and next permeabilized with 0,1% Triton X-100 (Sigma Aldrich). After that unspecific antibodies’ interactions were blocked with a 1% bovine serum albumin (BSA; Sigma Aldrich) solution. Next, primary antibodies directed against: α parvin (Cell Signalling, 8190 S, 1:200), cortactin (Santa Cruz Biotechnology, sc-11408, 1:100), YAP/TAZ (Cell Signalling, 8418 S, 1:100), β actin (Bio-Rad, MCA5775GA, 1:200), γ actin (Bio-Rad, MCA5776GA, 1:200) and then secondary antibodies (donkey anti-rabbit, Alexa Fluor 488, Invitrogen, A21206, dilution 1:200) were applied. Additionally, cells were treated with phalloidin-Alexa Fluor 568 (Thermo Fisher, 1:400) to stain filamentous actin (F-actin). To detect monomeric actin (G-actin), Alexa Fluor™ 594-labeled DNase I (Invitrogen, D12372, dilution 1:100) was used. 1000-times diluted Hoechst 33,342 reagent (Invitrogen) was applied to stain cell nuclei. To visualize cell shape, HCS CellMask™ Stain was used (Invitrogen, H32721, dilution 1:10000). Image acquisition was performed employing a Leica Stellaris microscope or an Opera Phenix confocal microscope (Perkin Elmer). Representative images for each condition are shown. At least three independent experiments were performed. Images obtained by Opera Phenix Plus with the use of a 63x (NA 1.15) water immersion objective were analyzed to determine the number, length, and width of stress fibers. Analysis was performed in FIJI software (1.54f) and a custom-written script. First, images were filtered with a Gaussian Blur filter (with Sigma set to 2 pixels) to remove noise. Next, stress fibers were detected by the “Ridge Detector” FIJI plug-in [10]. Sigma parameter was set to 3.96, minimum line length was set to 10, and the method for overlap resolution was set to “none”. Subsequently, all data were processed in R (version 4.3.1). The images used to detect stress fibers were also analyzed in FIJI to determine the number of invadopodia. Similarly, a script was used to automate analysis, and the next data were processed in R. The number, area, and length of focal adhesions and F: G actin ratio were analyzed utilizing the Perkin Helmer Harmony software v. 5.1.

### Cell adhesion assay

After seeding into the wells of 96-well plates, control and resistant cells were left to adhere for 1 h at 37 °C and washed a few times with PBS. Then, the XTT (2,3-Bis-(2-Methoxy-4-Nitro-5-Sulfophenyl)−2 H-Tetrazolium-5-Carboxanilide, disodium salt) test (Cell Proliferation Kit II; Roche) was performed to assess the number of cells adhering to the surface. After the addition of the XTT mixture, cells were incubated for 3 h at 37 °C. The absorbance of formazan was measured at 450 nm using a Quant spectrophotometer with Gen5 software (ver. 2.05). Three separate experiments were carried out, and all conditions were performed in five replicates. The number of adherent cells was calculated based on the absorbance as a percentage of the control (where the control is defined as 100%).

### Human protease array

A Proteome Profiler Human Protease Array Kit (R&D Systems, #ARY021B) was utilized to identify the components of the control and resistant melanoma cells’ secretome. Media utilized for the array were prepared as defined earlier [11]. The chemiluminescent signal was detected (using streptavidin-horseradish peroxidase), measured with a Bio-Rad ChemiDoc Imaging System, and then analyzed with Bio-Rad ImageLab software. After background correction, the densitometric signal was normalized to the mean of reference dots for each membrane.

### Gelatin zymography

The proteolytic activity of gelatinases in control and resistant culture media was detected based on gelatin zymography. Cell-conditioned media were prepared as defined earlier [11]. Next, the amount of protein was measured using a BCA procedure (Thermo Fisher), and media were separated on SDS-polyacrylamide gels containing gelatin (1 mg/ml). Then Coomassie Brilliant Blue R-250 (Sigma) was used to stain the gels. Brighter stripes on gels indicated the places where MMPs degraded the gelatin. Images were taken by ChemiDoc (Bio-Rad), and densitometric analysis was performed in ImageLab software from at least three biological repetitions. Representative gels are shown in Fig. 7.

### Matrix metalloproteinase 14 (MMP14) activity test

To assess the activity of MMP14, the SensoLyte 520 MMP14 Assay Kit (AnaSpec) was applied. Growing in 6-well plates cells were washed with PBS, collected in containing 0.1% Triton-X 100 assay buffer, and then incubated at 4 °C for 10 min. Next, samples were centrifuged (2500×g, 10 min, 4 °C), and the supernatants were placed into new tubes. The BCA assay was used to determine the protein concentration. The substrate was added to the samples containing the identical amount of protein (30 µg), previously incubated at 37 °C for 2.5 h with an activator. At 37 °C, the enzymatic reaction lasted for 30 min before being stopped by a stop solution. Next, using the GloMax Discover plate reader (Promega), the fluorescence of the product was measured (Ex/Em = 475/500 nm). In control samples, MMP14 activity was set at 100%. Three independent experiments were conducted in two replicates for every condition.

### Real-time PCR

To assess the expression level of chosen genes, RNA was isolated using a miRNeasy Mini Kit (Qiagen). Next, DNase I digestion with RNase-Free DNase Set (Qiagen) was applied, and then, using a High-Capacity cDNA Reverse Transcription Kit (Applied Biosystems), reverse transcription reaction was performed. All steps were carried out in accordance with the manufacturer’s protocols. PowerUp™ SYBR™ Green Master Mix was used for quantitative PCR on a StepOnePlus system (Applied Biosystems). Table 1 displays the primer sequences that were used. Based on the comparative CT (threshold cycle value) method (ΔCT = 2^- (CT gene of interest − CT housekeeping gene) results were normalized to *HPRT1* (hypoxanthine phosphoribosyltransferase 1) expression. At least three experiments were conducted, in duplicate for each sample.

**Table 1:** Primers’ sequences used for quantitative PCR analysis. List of abbreviations: *ADAM17/9* (a disintegrin and metalloproteinase 17/9), *TIMP1/2/3* (tissue inhibitor of metalloproteinases 1/2/3), *HPRT1* (hypoxanthine phosphoribosyltransferase 1)

Gene	Forward Primer 5’ – 3’	Reverse Primer 5’ – 3’
*ADAM17*	GATCATCGCTTCTACAGATACATG	CTTGAGAATGCGAATCTGCTC
*ADAM9*	GGCAATTGTGGTTTCTCTGG	GATCCTAGCTGGAAATCCACAC
*TIMP1*	GCTTCTGGCATCCTGTTGTTG	ACGCTGGTATAAGGTGGTCTG
*TIMP2*	GGTCAGTGAGAAGGAAGTGGAC	GGGGGCCGTGTAGATAAACTC
*TIMP3*	GCCTTCTGCAACTCCGACATC	CAGCTTAAGGCCACAGAGACTC
*HPRT1*	gaccagtcaacaggggacat	gcttgcgaccttgaccatct

### Statistical analysis

All data are presented as the means ± standard deviation (SD), and a Welch’s t-test was applied to assess their significance using GraphPad Prism 7 software.

## Results

**Fig. 1:**
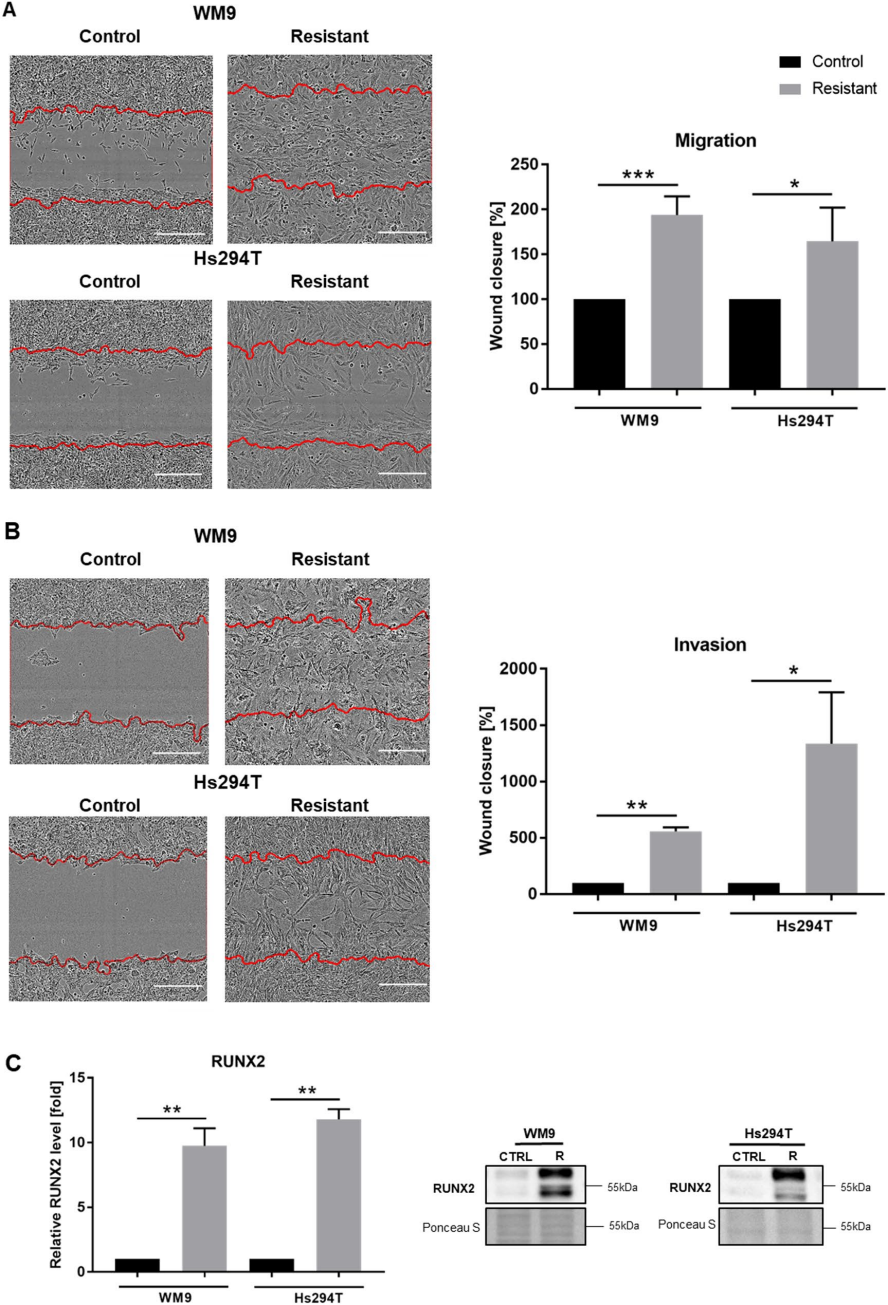
Migration and invasion of resistant melanoma cells. A scratch wound assay was used to evaluate the rate of migration (**A**) and invasion (**B**) of the investigated cells. Representative images of wounds covered by cells in 24 h (migration) or 48 h (invasion) are shown with red lines indicating the initial scratch area. The scale bar is set at 300 μm. The results’ quantification (**A**,** B**) is reported as the mean relative wound closure obtained from at least three independent tests ± SD. (**C)** Western Blotting analysis of RUNX2 level. Ponceau S staining was used to normalize the signal to the total protein content. The blotting membranes shown are representative of at least three independent biological replicates. WM9 and Hs294T cells treated with medium containing DMSO at the concentration used for drug delivery constitute the control (CTRL). Statistical significance was defined as *p* ≤ 0.05 (*), *p* ≤ 0.01 (**), and *p* ≤ 0.001 (***)

In our research, we have focused on melanoma cell invasion, which is driven by rearrangements in the actin cytoskeleton and matrix degradation. Proteolytic enzymes secreted by the cancer cells facilitate the cell migratory process to gain access to the blood vessels and spread. We used a scratch wound assay to assess the motility of resistant cells. This test evaluates the ability of cells to overgrow a previously made wound. To ensure conditions close to physiological ones, where melanoma cells migrate on the surface of the basement membrane or through elements of the extracellular matrix (ECM), the test was performed in the presence of Matrigel. It imitates the components of the basement membrane and is composed of elements of ECM [12]. For assay in 2D and 3D conditions (migration and invasion tests, respectively), cells were seeded into wells coated with Matrigel, while for the invasion assay, an additional layer of Matrigel was placed on top of the cells. We detected that both obtained BRAF/MEK inhibitors (BRAFi/MEKi) melanoma cell lines migrated (Fig. 1 A) and invaded (Fig. 1B) much faster compared to control lines. Resistant cells were able to almost completely close the scratch within 24 h (migration test) or 48 h (invasion test), whereas control cells only slightly closed the scratch after this time. Additionally, the increased motile abilities of resistant cells were more prominent in the invasion test. Moreover, we found that resistant cells retained decreased proliferation and greater migratory and invasive abilities even after drugs withdrawal for several weeks (Supplemental Fig. 1). In our previous work we showed that resistant cells expressed increased levels of growth factor receptors, including EGFR and PDGFRβ [7], which activate selected signaling pathways, such as the PIK3/AKT pathway, supporting cell invasion [13]. One of the factors that may contribute to the increased expression of the above-mentioned growth factor receptors and thus activation of AKT kinase in melanoma cells is the RUNX2 (Runt-related transcription factor 2) protein. We noted that in the studied cells, its level was increased in WM9 and Hs294T resistant cell lines in comparison to the control (Fig. 1 C), which may contribute to their increased invasive abilities.

**Fig. 2:**
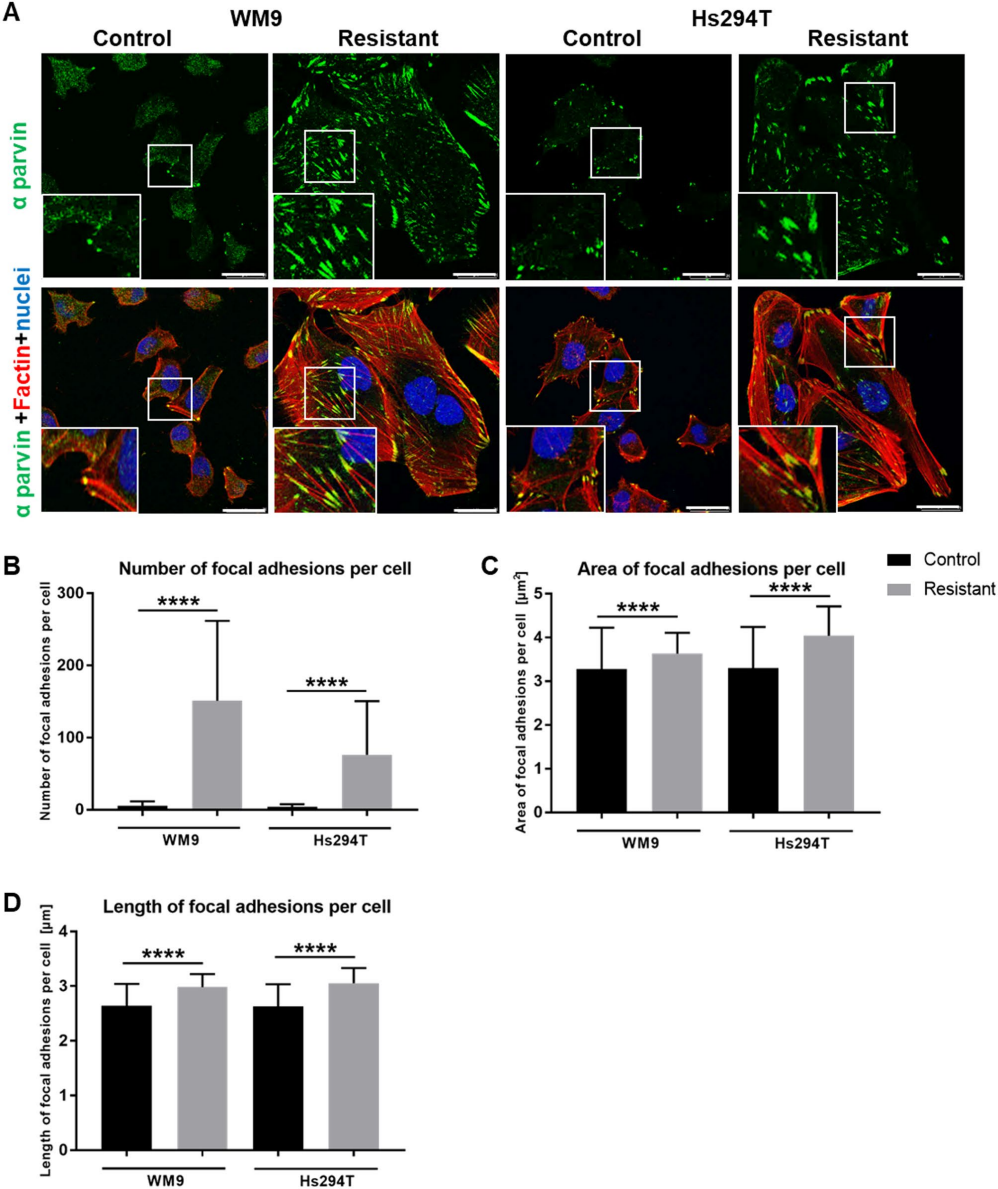
Focal adhesions’ parameters of control and resistant melanoma cells. **(A)** Representative images of control and resistant melanoma cells stained for α parvin (green), cell nuclei (blue), and F-actin (red). All labeled structures are visible on merged pictures. Enlargements of focal adhesion-rich areas (boxed) are shown as insets. Scale bar—25 μm. Focal adhesions’ number (**B**), area (**C**), and length (**D**) were calculated based on high-throughput microscope measurements. The analysis was performed for at least 2000 cells representing each condition and in three biological replicates. WM9 and Hs294T cells treated with medium containing DMSO at the concentration used for drug delivery constitute the control (CTRL). Data are presented as the average ± SD. Differences between control and tested cells are indicated by asterisks. Statistical significance was defined as *p* ≤ 0.0001 (****)

Due to the important role that adhesion plays during cell movement, we decided to determine the adhesive abilities of the examined cells at the next stage of the study. Cells were stained to visualize focal adhesions (FA) - structures linking the intracellular cytoskeleton to the ECM and thus participating in the development of cell-substrate interactions [14]. To visualize FA, we performed staining for α-parvin (Fig. 2 A), an adaptor protein that links the cytoskeleton and the integrin receptors [15]. Additionally, the actin filaments were stained with phalloidin, which binds to filamentous actin (F-actin), being a component of focal adhesions as well. During microscopic observation of cells, we noticed that resistant ones formed more focal adhesions, visible both on single α-parvin staining and on merged images, than control ones (Fig. 2 A; FA visible more precisely in enlargements of the FA-rich area (boxed)). Analysis of stained cells with high-throughput confocal microscope showed that WM9 and Hs294T cells resistant to treatment with BRAF/MEK inhibitors indeed formed a significantly higher number of focal adhesions (Fig. 2B). These structures also had a larger surface area (Fig. 2 C) and were longer than FA of the control cells (Fig. 2D).

**Fig. 3:**
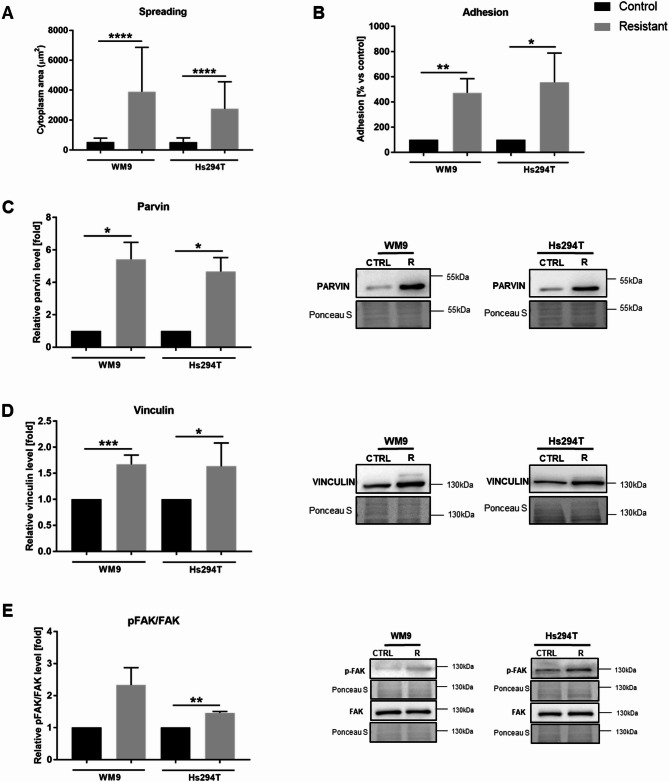
Spreading and adhesive properties of control and resistant melanoma cells. **(A)** Cytoplasm area (cell spreading) was measured based on images visualizing HCS CellMask™ labelling in tested cells using Harmony Opera Phoenix software. (**B)** The adhesion abilities of the examined cells. Levels of proteins related to cell adhesion: α parvin (**C**), vinculin (**D**), and pFAK/FAK ratio (**E**) were determined using Western blotting analysis. Ponceau S staining was used to normalize the signal to the total protein content. The blotting membranes shown are representative of at least three independent biological replicates. The graphs present average data ± SD from a minimum of three separate experiments. Asterisks indicate statistically important differences between the tested and control cells. WM9 and Hs294T cells treated with medium containing DMSO at the concentration used for drug delivery constitute the control (CTRL). Statistical significance was defined as *p* ≤ 0.05 (*), *p* ≤ 0.01 (**), *p* ≤ 0.001 (***), and *p* ≤ 0.001 (****)

Moreover, the cell surface area was also taken under examination, as the number of FAs determines the spreading of a cell [16]. Indeed, we noticed that resistant cells were significantly more spread, and confocal screening microscopy analysis confirmed our observations (Fig. 3 A). To verify whether resistant cells also adhere more strongly to the surface than control ones, we conducted cell adhesion tests. We observed that resistant cells exhibit approximately 5-fold greater adhesive capacity than control ones (Fig. 3B). Additionally, we assessed the level of proteins involved in the formation of focal adhesions, such as α-parvin and vinculin [17, 18], as well as those constituting elements of signaling pathways regulating this process like FAK (focal adhesion kinase), which also regulates tumor cells’ invasiveness by controlling focal adhesion-mediated cell motility [19]. Western blot analysis revealed increased levels of α parvin (Fig. 3 C) and vinculin (Fig. 3D) as well as elevated activation of FAK kinase (Fig. 3E) in resistant cells in comparison to control ones (pFAK/FAK ratio was statistically significant only for Hs294T cells).

**Fig. 4:**
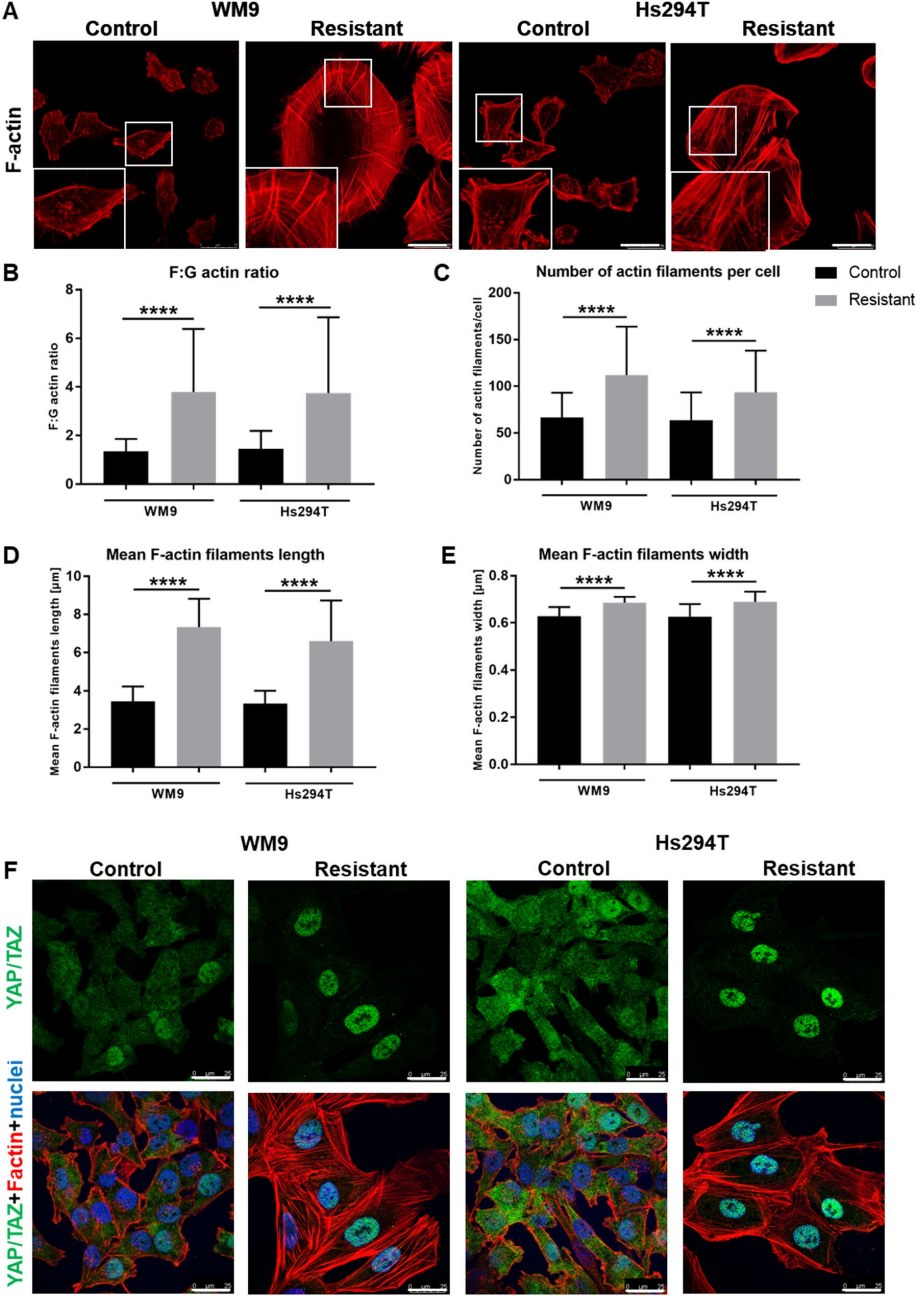
Differences in actin cytoskeleton organization between control and resistant melanoma cells. (A) Representative images of control and resistant melanoma cells stained for filamentous actin (F-actin; red). Enlargements of the stress fibers-rich area (boxed) are presented as insets. Scale bar—25 μm. (B) Filamentous to monomeric (F: G) actin ratio in tested cells, quantified using high throughput confocal microscope. Actin filaments parameters: number (C), length (D), and width (E) were calculated based on confocal images using ImageJ software. The analysis was performed for at least 200 cells representing each condition and for three biological replicates. F Representative images of control and resistant melanoma cells stained for YAP/TAZ (green), F-actin (red), and cell nuclei (blue). All labeled structures are visible on the merged pictures. Scale bar—25 μm. The graphs present average data ± SD from a minimum of three separate experiments. WM9 and Hs294T cells treated with medium containing DMSO at the concentration used for drug delivery constitute the control (CTRL). Asterisks indicate statistically important differences between the tested and control cells. Statistical significance was defined as p ≤ 0.0001 (****)

Since we observed a significantly increased spreading of resistant cells and an increase in the number of FA, we decided to check whether other changes in the organization of the actin cytoskeleton also occur in these cells. Quantitative analysis of microscopic images showed that the degree of actin polymerization, measured as the ratio of filamentous actin (F-actin) to monomeric actin (G-actin), is higher in resistant cells than in control ones (Fig. 4B). Microscopic images of cells stained for F-actin also illustrated that cells of both resistant lines form more prominent stress fibers (Fig. 4 A; stress fibers are visible precisely in enlargements of the boxed, stress fibers-rich area). Moreover, quantitative analysis of the observed stress fibers indicated that resistant cells formed significantly more of them (Fig. 4 C). Additionally, these fibers were characterized by increased length (Fig. 4D) and width (Fig. 4E) compared to the same structures formed by control cells. Moreover, it was demonstrated that increased actin cytoskeletal tension promotes YAP/TAZ nuclear translocation. These proteins are transcriptional coactivators and promote resistance to anti-cancer therapies [20]. We verified the localization of YAP/TAZ and noticed that in the case of control cells, they are localized throughout the cell body, whereas in resistant cells, they show nuclear localization (Fig. 4 F).

**Fig. 5:**
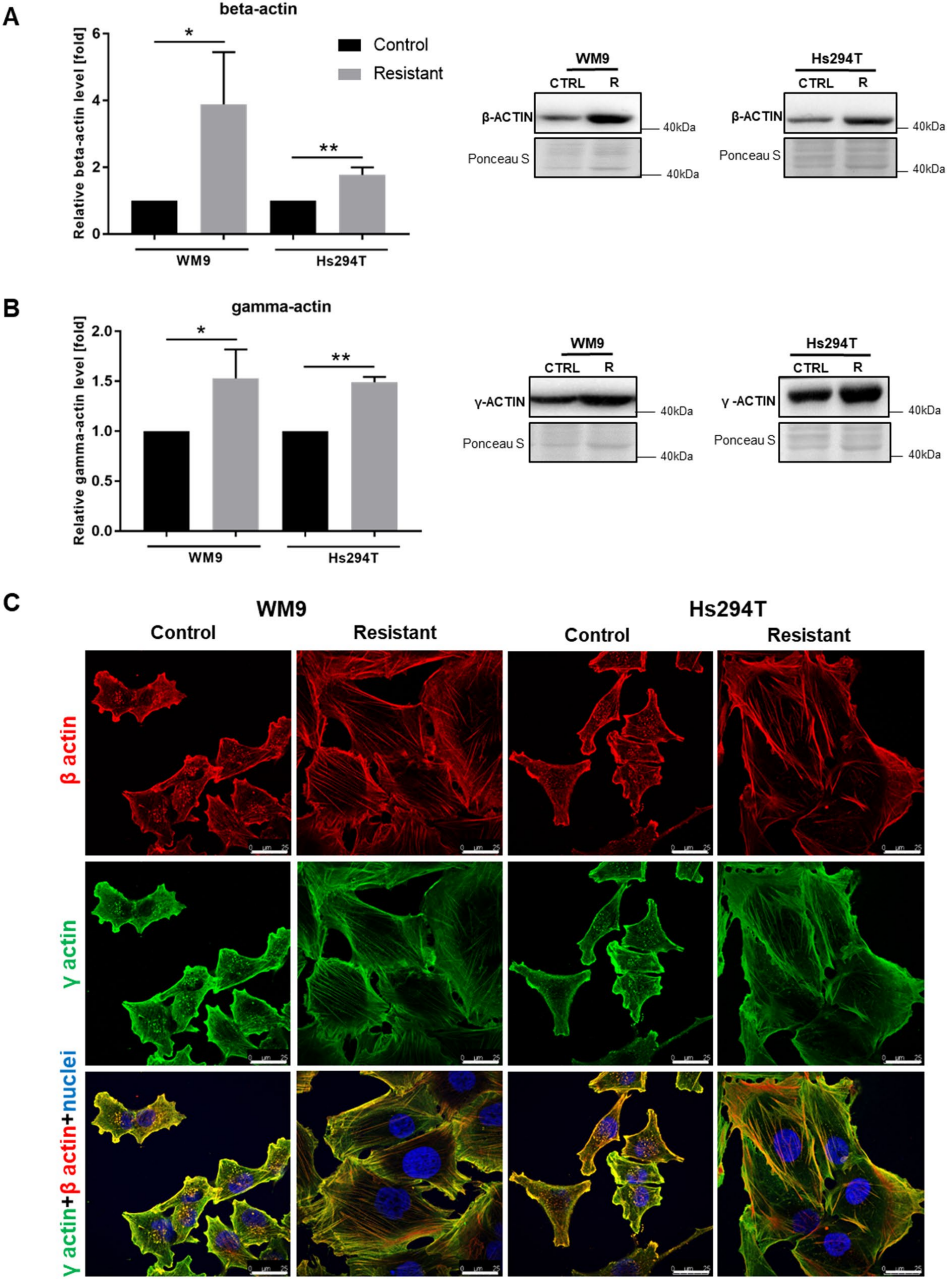
The level and localization of β and γ actin isoforms in control and resistant melanoma cells. The β (**A**) and γ (**B**) actin levels were determined by Western blotting analysis. Ponceau S staining was used to normalize the signal to the total protein content. The blotting membranes shown are representative of at least three independent biological replicates. The graphs present average data ± SD from a minimum of three separate experiments. WM9 and Hs294T cells treated with medium containing DMSO at the concentration used for drug delivery constitute the control (CTRL). Asterisks indicate statistically important differences between the tested and control cells. Statistical significance was defined as *p* ≤ 0.05 (*), *p* ≤ 0.01 (**). **(C)** Representative images of control and resistant melanoma cells stained for β (red) and γ (green) actin, and cell nuclei (blue). All labeled structures are visible on the merged pictures. Scale bar—25 μm

There is an ongoing discussion about the role of non-muscle actin isoforms (β and γ) in building individual cytoskeletal structures and in cell migration [21–24]. Thus, we assessed the level (Fig. 5 A, B) and cellular localization (Fig. 5 C) of β and γ actin. Western blot analysis demonstrated that the levels of both isoforms were increased in resistant cells compared to the control ones (Fig. 5 A, B). Immunocytochemical staining has shown that both isoforms are located in all actin structures formed by cells, including stress fibers, lamellipodia, and invadopodia (Fig. 5 C).

**Fig. 6:**
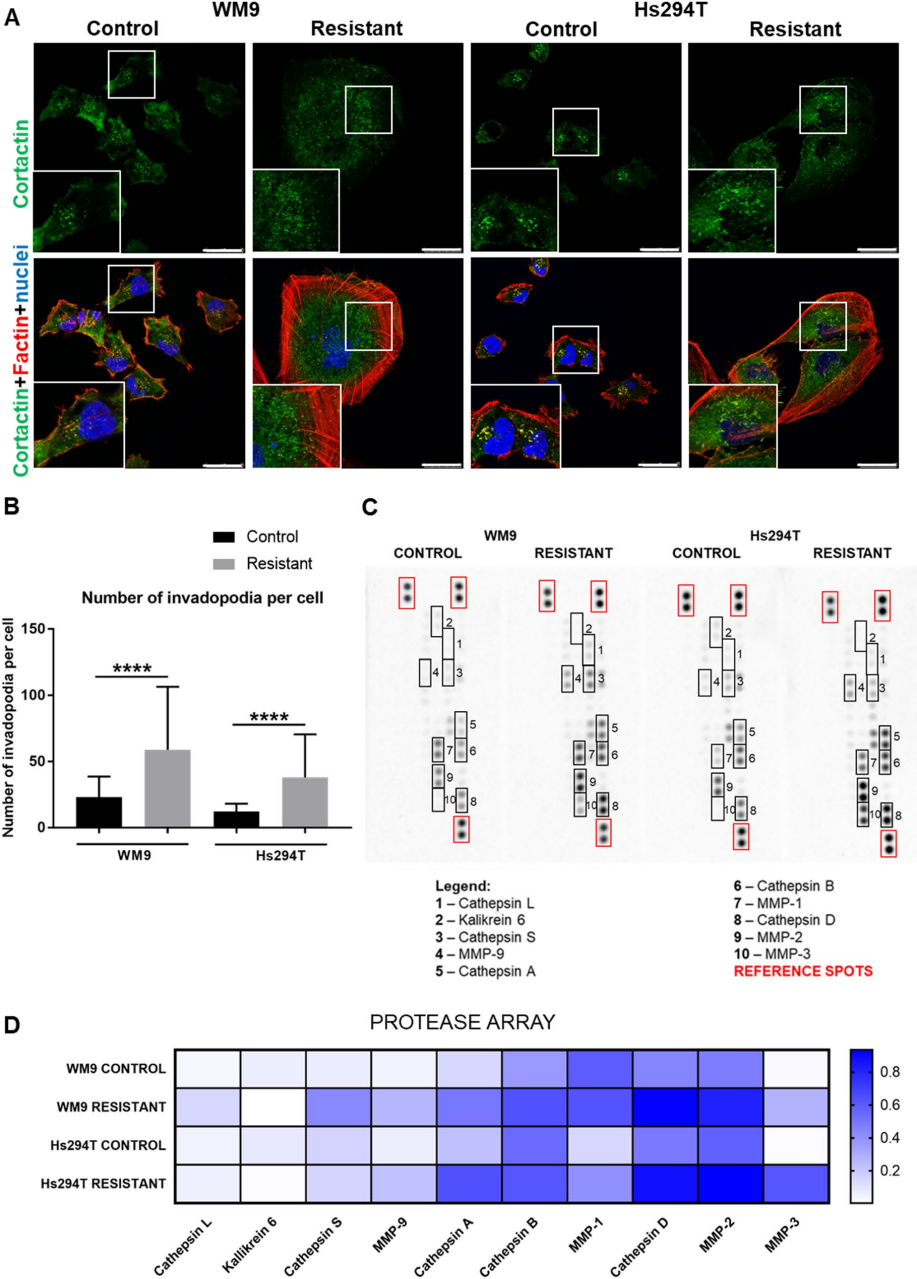
Invadopodia formation and profile of proteases secreted by resistant melanoma cells.** (A)** Representative images of control and resistant melanoma cells stained for cortactin (green), cell nuclei (blue), and F-actin (red). All labeled structures are visible on merged pictures. Enlargements of the invadopodia-rich area (boxed) are presented as insets. Scale bar—25 μm. **(B)** The number of invadopodia was calculated based on cell staining with anti-cortactin antibody for at least 200 cells representing each condition and three biological replicates. The graphs present average data ± SD from a minimum of three separate experiments. Statistical significance was defined as *p* ≤ 0.0001 (****), and is indicated in the graphs by asterisks. The proteases array served to identify secreted proteases by cells in conditioned media (**C**). Quantitative analysis was performed based on the obtained signals. Normalized to reference spots, results were presented in the form of a heatmap, where a higher signal intensity is indicated by darker blue. **(D)** WM9 and Hs294T cells treated with medium containing DMSO at the concentration used for drug delivery constitute the control (CTRL). Abbreviations: MMPs, matrix metalloproteinases

Mesenchymally migrating cells, like melanoma cells described here, form specialized structures called invadopodia during the invasion process. These are actin-rich protrusions exhibiting proteolytic activity towards the substrate [25]. Among molecular components of invadopodia, we can distinguish cortactin and F-actin, which are the most common proteins of these structures [26]. We have stained and examined melanoma cells for the presence of cortactin and F-actin to detect the occurrence of invadopodia in these cells (Fig. 6 A; invadopodia are visible precisely in enlargements of the boxed, invadopodia-rich area). Resistant cells form more of the mentioned protrusions than control ones (Fig. 6B). Invadopodia can secrete matrix metalloproteinases (MMPs) and degrade many variable elements of ECM, including collagen types I and IV, laminin, and fibronectin [27]. Moreover, invasion is largely dependent on the pathways involved in processes occurring in dense extracellular matrix; thus, mesenchymally migrating cells often secrete proteases, which degrade proteins present in the cellular microenvironment [28]. That is why we decided to verify the proteolytic abilities of cells resistant to the combined BRAFi/MEKi therapy. The Proteome Profiler Human Protease Array allowed us to identify the elements of control and resistant melanoma cells’ secretome. We found several differences in the secretion profile of the examined cells (Fig. 6 C, D). In resistant melanoma cell lines, we identified increased levels of cathepsins: A, B, D, L, S and matrix metalloproteinases (MMPs): MMP1, MMP2, MMP3, MMP9, while the level of kallikrein 6 was slightly decreased.

**Fig. 7:**
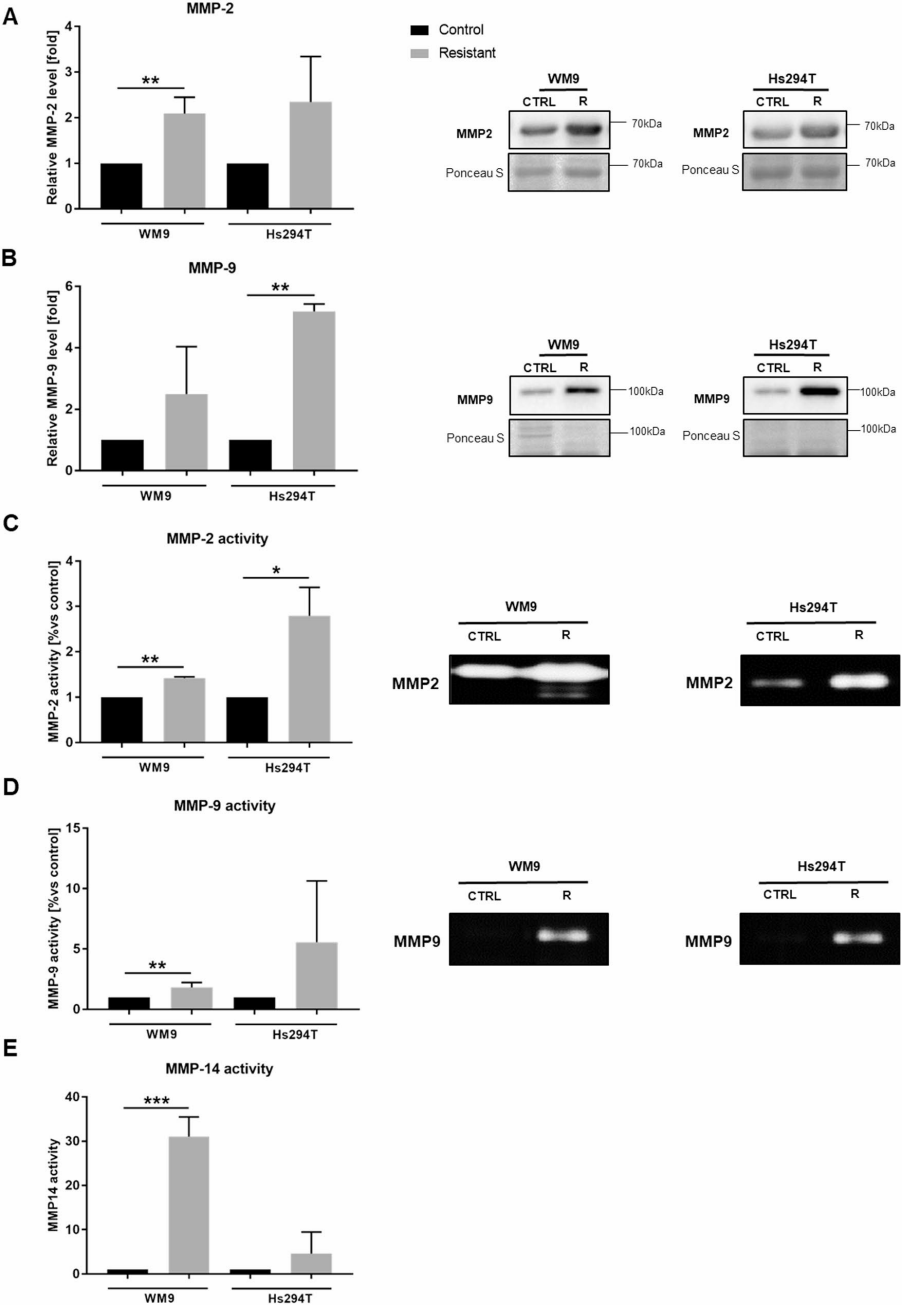
Matrix metalloproteinases’ level and activity in resistant melanoma cells. Western Blotting analysis of MMP2 (**A**) and MMP9 (**B**) levels in cell-conditioned media derived from control and resistant melanoma cells. MMP2 (**C**) and MMP9 (**D**) activity were detected using gelatin zymography analysis of media collected from examined cells. **(E)** MMP14 activity assay was performed on cell lysates. WM9 and Hs294T cells treated with medium containing DMSO at the concentration used for drug delivery constitute the control (CTRL). Ponceau S staining was used to normalize the signal to the total protein content. Blotting membranes and zymography gels shown are representative of at least three independent biological replicates. Results are presented as the mean ± SD of a minimum of three separate experiments. Asterisks indicate statistically important differences between the tested and control cells. Statistical significance was defined as *p* ≤ 0.05 (*), *p* ≤ 0.01 (**), and *p* ≤ 0.001 (***). Abbreviations: MMPs, matrix metalloproteinases

The quantity and diversity of proteases detected in the protease array suggested us to analyze these cells’ proteolytic activity further. At first, we verified the level of MMP2 and MMP9 in cell-conditioned media collected from control and resistant melanoma cells. We noticed elevated levels of both of these enzymes in resistant cells (Fig. 7 A, B). Next, we used a gelatin zymography to estimate the activity of secreted gelatinases in resistant cells (Fig. 7 C, D). We found out that both WM9 and Hs294T resistant cells demonstrate an increased level of active MMP2 as well as MMP9; however, no statistical significance was observed for MMP9 activity in the resistant Hs294T cell line. Other metalloproteinases, which are not secretory enzymes but membrane ones, are also capable of degrading the surrounding matrix. Collagenase MMP14 (MT1-MMP) is a key enzyme in this group, primarily degrading type I collagen [29]. Fluorimetric assays of whole-cell lysates revealed significantly increased MMP14 activity in both resistant cell lines compared to control ones, whereas statistical significance was limited to the WM9 cells (Fig. 7E).

**Fig. 8:**
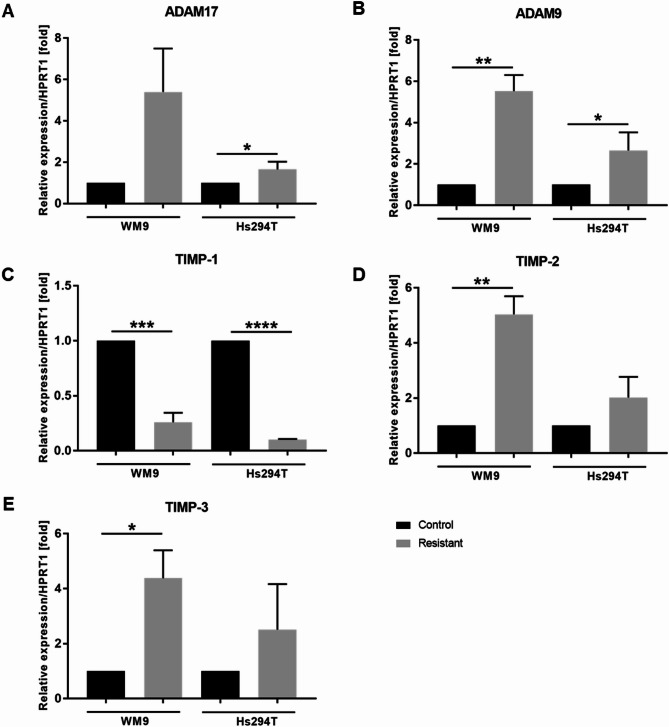
Expression level of genes associated with proteolysis in melanoma cells resistant to BRAFi/MEKi. (**A)** *ADAM17*, (**B**) *ADAM9*, (**C**) *TIMP1*, (**D**) *TIMP2*, and (**E**) *TIMP3* mRNA levels in control and resistant melanoma cells. HPRT1 served as the reference gene for Real-Time PCR analysis. WM9 and Hs294T cells treated with medium containing DMSO at the concentration used for drug delivery constitute the control (CTRL). The graphs present average data ± SD from a minimum of three separate experiments. Asterisks indicate statistically important differences between tested and control cells at the level of *p* ≤ 0.05 (*), *p* ≤ 0.01 (**), *p* ≤ 0.001 (***), and *p* ≤ 0.0001 (****). Abbreviations: ADAM, a disintegrin and metalloproteinase; TIMP, the tissue inhibitor of metalloproteinases

In addition, other proteases may also be involved in the digestion of ECM components. We noticed that resistant melanoma cells exhibit increased expression levels of genes encoding proteases from the ADAM family (disintegrin and metalloproteinase domain-containing proteins), including *ADAM9* and *ADAM17* (Fig. 8 A, B; no statistical significance for the *ADAM17* gene in the WM9 cell line). On the other hand, proteolytic regulation involves not only protease levels but also the expression of their inhibitors like tissue inhibitors of metalloproteinases (TIMPs). They usually inhibit protease activity and thus cancer cell invasion, but in certain situations, they can also support cancer progression [30, 31]. We have verified the expression level of genes encoding TIMPs in the examined cells. Resistant cells exhibited downregulation of *TIMP1* gene expression compared to control ones (Fig. 8 C), while, interestingly, the expression levels of *TIMP2* and *TIMP3* (Fig. 8D, E) were elevated in these cells. The aforementioned changes were not statistically significant for the Hs294T cell line for the *TIMP2* and *TIMP3* genes.

Since the invasion of resistant cells is a major clinical problem, we attempted to inhibit this process in the aforementioned cells by using several inhibitors blocking the activity of proteins that are key to invasion and resistance, such as EGFR, MET, AKT, metalloproteinases, and ABCA1. Unfortunately, in most cases, applied compounds reduced the invasive capacity of only one of the tested lines or only slightly (by 20–30%) (Supplemental Fig. 2).

## Discussion

In our previous work, we preliminarily characterized melanoma cells resistant to treatment with vemurafenib (BRAFi) and cobimetinib (MEKi) [7]. In the current studies, we characterized double-resistant cells in terms of their capacity to migrate and invade, as these processes may lead to the formation of in vivo metastases, which are the main cause of death in patients suffering from melanoma. Our analysis of the motility of resistant cells showed they had a significantly greater ability to migrate (2D conditions) and invade (3D conditions) than control cells. Other researchers have made similar observations [32–34]. Patel et al. demonstrated remarkably elevated invasive properties in three encorafenib/binimetinib-resistant cell lines in comparison to the naïve cell lines [32]. Moreover, the increased invasive capacity of resistant cells has been demonstrated using several research models. Vultur et al., utilizing skin reconstructs, indicated that MEK-inhibitor-resistant melanoma cells invaded deeper into the dermis than control cells [35]. Paraiso et al. showed that BRAFi/MEKi-resistant cells presented elevated motile behavior in transendothelial migration assays and spheroid assays [34].

As we indicated before, examined resistant cells demonstrated a lower proliferation rate [7], which, combined with increased invasion abilities, suggests that they undergo the phenomenon called “phenotype switching”. This process of changing the state of cells from proliferative to invasive allows cancer cells to become resistant to treatment [36]. In the case of examined cells, this phenotype switch is permanent, as after withdrawal of the drugs, after several passages, the resistant cells still present reduced proliferation and increased migration/invasion in comparison to control cells. Most often, the phenotype-switching process is accompanied by the appearance of epithelial-mesenchymal transition (EMT) features in resistant cells [32, 34, 37]. We also observed earlier that melanoma cells resistant to vemurafenib and cobimetinib are characterized by increased mRNA level of *SLUG* (SNAI2; snail family transcriptional repressor 2) and elevated level of transforming growth factor β receptors: TGFβRI and TGFβRIII, with a simultaneous decrease in *SOX10* (SRY-box transcription factor 10) expression, which contributes to the induction of EMT and reduction of cell proliferation [7, 33, 38, 39]. This less differentiated phenotype is frequently associated with elevated levels of receptor tyrosine kinases and elements of signaling pathways such as PDGFRβ, EGFR, ERK, AKT, JNK, and p38 [13, 40–43]. We also detected elevated expression of receptors: *EGFR*, *ErbB2 (erb-b2 receptor tyrosine kinase 2)*, *MET*, *PDGFRβ*, and activation of signaling pathways manifesting as increased pAKT/AKT, p-p38/p38, pJNK/JNK ratios in vemurafenib/cobimetinib resistant cells [7]. All of these factors may regulate cell migration and invasion, but by distinct mechanisms. Previous studies indicated that the MAPK pathway is essential for cell migration. The MAPK family can be divided into three groups: extracellular-signal-regulated protein kinase (ERK/MAPK), p38, and Jun N-terminal kinase (JNK) [43]. p38 modulates migration by phosphorylating MAPK-activated protein kinase 2/3 (MAPKAP 2/3), which appears to be significant for the directionality of migration [41]. JNK regulates cell migration by phosphorylation of paxillin, Jun, and microtubule-associated proteins [41]. AKT leads to transcriptional changes in EMT-related genes such as E-cadherin, ZEB1 (zinc finger E-box binding homeobox 1), and Snail (SNAI1; snail family transcriptional repressor 1) [13]. The factor whose level is elevated in the WM9 and Hs294T resistant cells and which also contributes to the increase in their migration capacity is RUNX2. This protein was described as a factor regulating the expression of genes intimately associated with cytoskeleton remodeling and proteolysis, and thus cellular movement. It also stimulates the PI3K/AKT pathway, whose role in EMT has been documented [44–46]. Moreover, melanoma cells resistant to the BRAF inhibitor exhibit significant upregulation of RUNX2, which is associated with an increase in EGFR level and phosphorylation. On the other hand, RUNX2 knockdown resulted in a reduction of EGFR and PDGFRβ levels [47]. Additionally, AKT can activate RUNX2 and thus contribute to RUNX2 transcriptional activity. Reciprocally, the stimulation of the AKT pathway by RUNX2 was also described. This mutual activation in the context of cancer cells presenting AKT phosphorylation and high levels of RUNX2 might constitute a major driving force in tumor progression and invasion [48].

In comparison to control, we have observed morphological changes in the cells resistant to treatment with BRAFi/MEKi. Examined cells were elongated, highly spread, and spindle-shaped. Similar to our observations, Dratkiewicz et al., Patel et al., and Kim et al. [19, 20, 49] also noted morphological changes in single drug resistant melanoma cells. It was previously demonstrated that the acquisition of the mesenchymal-like state is connected to a great cytoskeletal rearrangement reflected by morphological changes with cells presenting a flattened and spindle-like shape [33]. Cell spreading and migration are regulated by FA, which are sites of cell-matrix interaction that constantly change or reorganize in response to microenvironmental cues [18, 50, 51]. We detected an increased number, area, and length of focal adhesions in both double-resistant cell lines, as well as an elevated adhesion abilities of these cells. Diazzi et al., utilizing phospho-paxillin staining, also noticed an elevated number of focal adhesions upon BRAF pathway inhibition [33]. Additionally, it was shown that exposure of melanoma cells to BRAF and MEK inhibitors results in increased expression of FA-related genes [52]. Our Western blotting analysis demonstrated raised levels of proteins that are elements of focal adhesions or regulate their assembly and disassembly, namely α-parvin, vinculin, and pFAK/FAK ratio. Proteins such as paxillin, FAK, vinculin, and α-parvin are recruited upon contacts between the ECM and the cell, which results in additional F-actin and focal adhesions formation [17]. Vinculin is a key regulator of FAs. Cells depleted of this protein exhibit reduced adhesion to ECM proteins, and fewer and smaller adhesions compared to wild-type cells [18]. As a component of FAs α parvin plays a role in cell adhesion, spreading, and motility [17]. FAK is a key focal adhesion signaling protein involved in FA turnover and cell spreading [53]. Phosphoproteomic studies demonstrated that chronic BRAF inhibition was associated with an enrichment of phospho-proteins involved in adhesion and cytoskeletal remodeling, including focal adhesion kinase [34]. Additionally, FAK is highly expressed in multiple tumors and correlates with their aggressiveness. It promotes tumor progression by altering cell invasion abilities, epithelial-mesenchymal transition, the tumor microenvironment, and the stemness of cancer cells. FAK mediates downstream activation of ERK and PI3K/AKT signaling pathways, enhancing cancer cell invasion [54–56]. We have previously shown that vemurafenib/cobimetinib-resistant melanoma cells exhibit elevated expression or level of cancer stem cells (CSC) markers (signal transducer CD24, Homeobox protein Nanog, activated leukocyte cell adhesion molecule - ALCAM) as well as increased activation of AKT in relation to sensitive cells [7]. FAK level is also associated with the RUNX2 level. It was demonstrated that RUNX2 knockdown in melanoma cell lines significantly decreased FAK expression and inhibited the growth and invasion of these cells [44].

In the turnover of focal adhesions may be also involved the above-mentioned JNK kinase, whose active form is localized in FAs. It was indicated that paxillin, one of the key components of these structures, is also a JNK substrate. Huang et al. speculate that phosphorylation of paxillin by JNK may stimulate its degradation and focal adhesion disassembly, and thus promote cell migration. The factors phosphorylating paxillin and possibly contributing to the turnover of FAs are also active forms of p38 and ERK kinases [41].

Changes involving the rearrangement of the actin cytoskeleton are also inextricably linked to cell migration and adhesion. We observed that cells resistant to vemurafenib and cobimetinib are characterized by increased actin polymerization (measured as F: G ratio) and increased number, length, and width of stress fibers. Stress fibers, which consist of about 10–30 microfilaments aligned in parallel, are partly responsible for cell contraction and adhesion [21]. Murali showed that the dabrafenib/trametinib-resistant cells display thick F-actin fibers across the entire cytoplasm [57]. Foda et al. also noticed that resistant to vemurafenib mouse melanoma cells exhibited increased stress fibers formation in vitro, while in vivo they developed more aggressive tumors, which resulted in a reduced lifespan of these mice [58]. It suggests that elevated stress fiber formation may be associated with increased aggressiveness of cancer cells. Wen et al. also demonstrated the correlation between stress fibers formation and the invasive abilities of melanoma cells. The two most invasive melanoma cell lines formed the highest number of stress fibers, the next one (slightly less invasive) had fewer but thicker stress fibers, and the last one had no stress fibers and was characterized by a lack of ability to move [59].

YAP/TAZ proteins are also involved in the alterations within the actin cytoskeleton in cells resistant to BRAF/MEK treatment. It was shown that inhibition of actin polymerization and actomyosin tension in melanoma cells suppresses both YAP/TAZ activation and BRAFi resistance. An increase in both actin stress fibers number and cytoskeletal tension is the key inducer of YAP/TAZ nuclear localization and its activation in BRAFi-resistant melanoma cells. Kim et al. demonstrated that resistant to BRAFi melanoma WM3248 cells exhibited an increase in nuclear YAP/TAZ localization when compared with parental cells [20]. We also noticed this phenomenon in double-resistant BRAFi/MEKi cells, which were characterized by a much more nuclear localization of the YAP/TAZ proteins compared to control cells. Actin-dependent YAP/TAZ activation plays a critical role in BRAFi resistance, also by promoting upregulation of EGFR and AKT. Moreover, YAP and TAZ are coactivators of transcription factors, such as mentioned earlier RUNX2. YAP activation has also been reported to be associated with cell stemness [20, 60].

The cytoskeleton of non-muscle cells, which undergoes intensive reorganization during cell movement, is built by two actin isoforms- β and γ. There has been a long-standing discussion regarding the role of both of these isoforms in the process of cell migration and adhesion. Our previous studies show that both of them play an analogous role during cell migration – they equally build stress fibers as well as migratory protrusions such as lamellipodia or invadopodia [22, 24]. Other researchers have shown, however, that γ actin is more involved in the process of cell migration and invasion than β actin [21, 23]. Due to these doubts, we determined the level and localization of both isoforms of actin in the resistant and sensitive cells. To our knowledge, data on the level and localization of beta and gamma actin in double-resistant melanoma cells have not been reported so far. We observed that the level of both β and γ actin is elevated in BRAFi/MEKi resistant cells. Moreover, both isoforms are analogously distributed in these cells, also in migratory protrusions, which may indicate their involvement in the process of cell invasiveness.

Other structures built by F-actin and involved in the invasion of cancer cells are invadopodia. These protrusions consist of an actin-rich core, which contains regulators of actin polymerization, and is surrounded by the proteins involved in adhesion, signaling, and scaffolding. Secreted by invadopodia, metalloproteinases play a key role in the proteolytic degradation of the ECM, contributing to cell adhesion and invasion. BRAF-mutated melanoma cells form a lot of invadopodia as ERK leads to phosphorylation of one of the invadopodia components - cortactin, which in turn regulates actin assembly and MMPs secretion [61]. We have noticed that double-resistant melanoma cells form an increased number of invadopodia in comparison to control cells. Shen et al. also demonstrated that vemurafenib triggered invadopodia formation. They also indicated that activation of Pyk2 was crucial for this process [62].

Upon maturation, invadopodia recruit proteases such as membrane type 1- matrix metalloproteinase (MT1-MMP), MMP2, and MMP9 [62, 63]. For this reason, in the next stage of our research, we thoroughly analyzed the proteolytic abilities of the examined cells. Melanoma cells resistant to vemurafenib/cobimetinib secreted into the culture medium an increased amount of several cathepsins (A, B, D, L, S) as well as matrix metalloproteinases (MMP1, MMP2, MMP3, MMP9) compared to the control. Cathepsins are proteases important for melanoma progression and therapeutic resistance. Extracellular cathepsins directly cleave the ECM and activate pro-invasive proteases [64]. Previous studies have shown that secretion of cathepsins B, L, D, A, and S is markedly increased in metastatic melanoma [64–67]. MMP-1 is highly expressed in invasive melanomas, contributing to cancer cell invasion by degradation of type I collagen [68, 69]. In vivo studies also demonstrated that MMP3 promotes melanoma tumor growth and lung metastasis [70]. In contrast to the previously mentioned enzymes, the kallikrein 6 level was slightly reduced in the examined resistant cells compared to controls. Pampalakis et al. showed that expression of this protein in melanoma cells results in markedly suppressed growth of primary tumors [71]. According to our knowledge, the involvement of any of the above-described proteases: cathepsins, MMP1, MMP3, and kallikrein 6 in the proteolysis carried out by resistant melanoma cells has not been previously described.

In the next stage of our work, we decided to investigate in more detail the involvement in proteolysis of those proteases that are associated with invadopodia, i.e., MMP2, MMP9, and MMP14 (MT1-MMP). We found that the level and activity of all of these enzymes were increased in resistant cells compared to the control ones. The expression of these enzymes is promoted by FAK and JNK [56]. MT1-MMP level is also upregulated in BRAFi-resistant cells [56, 72]. Upon BRAFi treatment, MMP2 is the main secreted metalloproteinase acting downstream of MT1-MMP, which directly activates it [56]. In addition to proteases, the proteolysis process is also regulated by TIMPs, which usually inhibit the activity of proteases. TIMP1 preferentially inhibits MMPs 7, 9, 1, and 3, whereas TIMP2 also inhibits MMP2. TIMP3 can inhibit MMP2 and MMP9 as well as most of the ADAMs [31]. As expected, the expression level of *TIMP1* in the tested resistant cells was reduced compared to the control ones. However, surprisingly, *TIMP2* and *TIMP3* were upregulated in comparison to the naïve cells. As far as we know, no one else has described similar results for TIMPs in resistant melanoma cells. It was demonstrated earlier that activation of proMMP2 can occur through the formation of a trimolecular complex between MMP14, TIMP2, and proMMP2 at the cell surface. TIMP2 was shown to be required for efficient proMMP-2 activation. This molecule is unique because it can act both as an MMP inhibitor and activator [31, 73, 74]. It was also shown that TIMP2 overexpression can protect human melanoma cells from apoptosis [75]. TIMP3 usually exerts antitumor effects via matrix metalloproteinase (MMP)-dependent pathways; however, conflicting reports indicate that expression of TIMP3 in cancers is higher than in healthy tissues [76, 77].

Since ADAMs proteases were not tested in the protease array, we verified their expression by RT-PCR and detected the increased expression of genes encoding *ADAM9* and *ADAM17* in resistant cells compared to the control ones. ADAMs are structurally highly related to matrix metalloproteinases. It was demonstrated earlier that high expression of ADAM9 is associated with human melanoma progression, to which it contributes through the cleavage of the laminin beta 3 chain, which is crucial in the process of invasion and metastasis formation [72]. Using immunohistochemistry, it was also demonstrated that ADAM17 is highly expressed in melanoma cells [78]. The presence of both proteins has not been previously described in treatment-resistant melanoma cells.

As mentioned earlier, applied by us compounds including EGFR, MET, AKT, metalloproteinases, and ABCA1 inhibitors decreased the invasive abilities only slightly (by 20–30%) or they affected only one of the tested lines. Moreover, thanks to parallel transcriptomic (RNA-seq analysis) and proteomic studies (gel-free nanoLC–MS/MS shotgun proteomic approach) (manuscripts under review), we have determined that both HS294T and WM9-resistant cells exhibit altered expression of approximately 800–900 genes/proteins compared to controls. Many of them are involved in the regulation of the organization of the actin cytoskeleton, proteolysis, migration, and invasion.

**Fig. 9:**
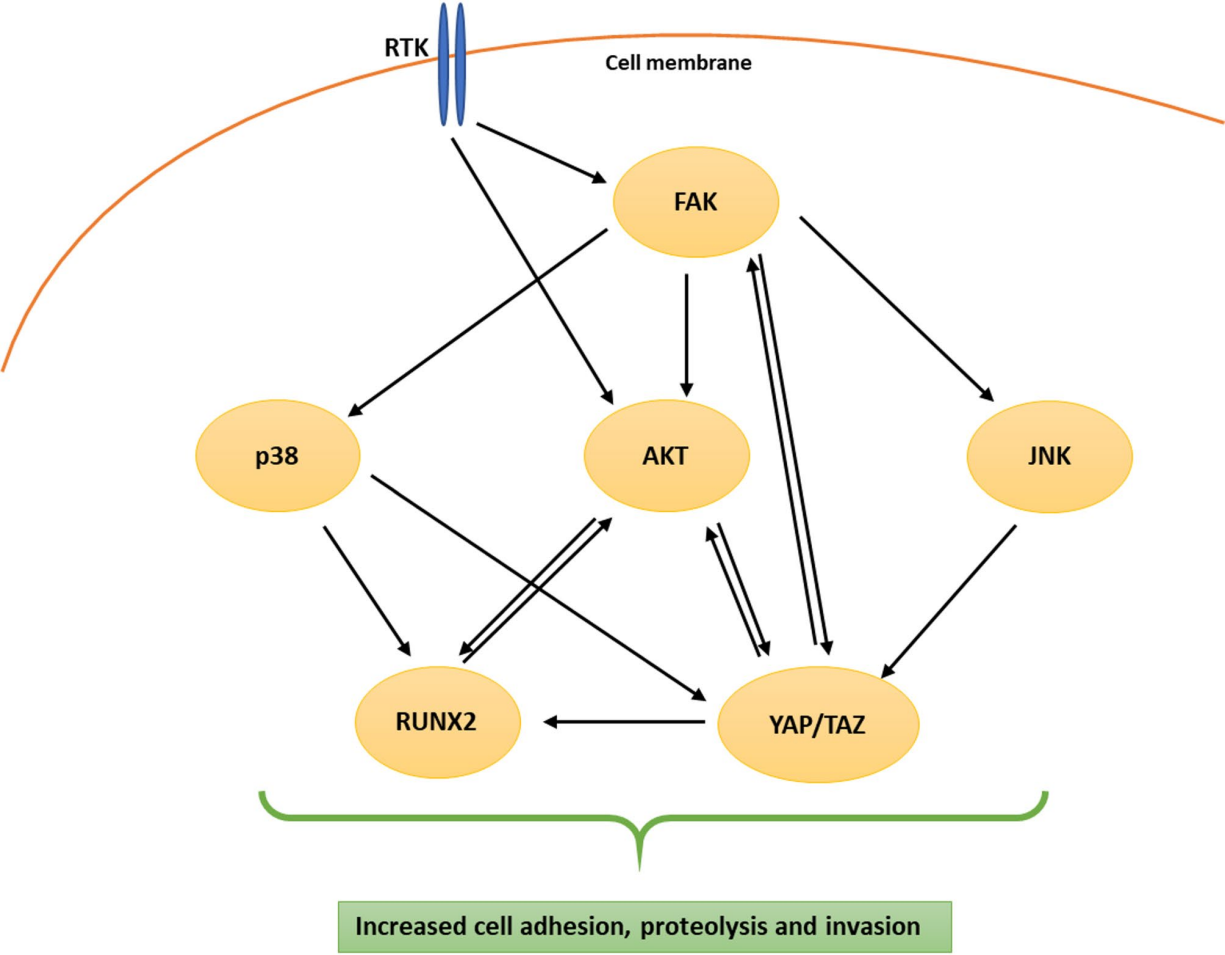
Mechanisms of increased invasive capacity of BRAF/MEK inhibitor-resistant melanoma cells presented as the network of mutually interacting proteins. Abbreviations: RTK – receptor tyrosine kinases, FAK - focal adhesion kinase, YAP - Yes-associated protein, TAZ - transcriptional coactivator with PDZ‐binding motif, p38- p38 mitogen-activated protein kinase, JNK- c-Jun N-terminal kinase, AKT- protein kinase B, RUNX2- runt-related transcription factor 2

The achieved results encourage us to reach out the hypothesis that the increased invasive capacity of resistant cells is not due to an alteration of a single protein level/activation, but rather to a network of interactions between multiple proteins that leads to the activation of signaling pathways, inducing the invasion process. The molecules comprising this network, whose levels are elevated (or localization is changed, as for YAP/TAZ) in the resistant cells studied, include receptor tyrosine kinases (RTK) (EGFR, MET, PDGFRβ), FAK, p38, JNK, and AKT kinases, as well as the RUNX2 and YAP/TAZ proteins (Fig. 9).

As shown in Fig. 9, the extracellular signal transmitted by RTK activates AKT and FAK kinases [79]. FAK mediates downstream activation of AKT, JNK, and p38 signaling pathways, enhancing cancer cell invasion by modulation of cell migration, adhesion, and proteolysis [41, 80–83]. Moreover, in some cases, e.g., in cholangiocarcinoma, FAK and activated AKT synergize to induce the YAP oncogene. FAK also plays a crucial role in YAP phosphorylation, which enhances its stability and facilitates nuclear translocation [84]. Interestingly, YAP also may activate FAK, supporting cancer cell migration as well as chemotherapy resistance, and cancer relapse [84]. Additionally, YAP/TAZ activation promotes upregulation of AKT. In the regulation of the Hippo signaling pathway that mediates the activity of co-transcriptional factors, YAP and TAZ are also involved JNK and p38 kinases [85, 86]. Moreover, YAP and TAZ are coactivators of additional transcription factors, such as RUNX2. Simultaneously, AKT can activate RUNX2, and vice versa [87–90]. These mutual interactions may contribute to the pathological properties of metastatic cells, similar to the stimulatory influence exerted on RUNX2 expression by the phosphorylated form of p38 kinase present in cancer cells [91, 92]. All the interactions described above have a stimulating effect on processes such as adhesion, migration, and proteolysis, which are integral parts of cell invasion.

The discussed above complex network of connections between proteins regulating cell motility is a main reason why developing an effective therapy targeting the proteins involved in the invasion of resistant cells is challenging. Blocking the signal transmitted from one protein may involve compensation by other proteins that activate the same downstream targets. Similar reports have been described in the literature previously. It was shown that FAK activation contributing to the activation of AKT or YAP may be one of the reasons for the low effectiveness of therapies directed against BRAF or MEK [84, 93]. Moreover, Riordan et al. showed that the drug resistance mechanism in BRAF-mutant melanoma may be based on Rac1 signaling, which drives the resistance, simultaneously activating YAP/TAZ, JNK, and p38 pathways [94]. Blocking so many proteins involved in the key cellular processes would be impossible in a clinical setting, because they are also important for the proper functioning of normal cells present in the patient’s organism. Selecting these few molecular targets whose simultaneous blocking could significantly reduce the invasion of melanoma cells resistant to therapy with BRAF/MEK inhibitors, without significantly disturbing the functioning of normal cells, is a challenge for the future.

In summary, melanoma cells resistant to vemurafenib and cobimetinib present a significantly greater ability to migrate and invade than control cells, which, in combination with the features of these cells described by us earlier, such as a lower proliferation rate, elevated levels of EMT and CSC markers as well as increased activation of selected signaling pathways, suggests that they may undergo phenotype switching. This phenomenon is associated with an extensive cytoskeletal rearrangement consisting of the formation of a greater amount of focal adhesions, invadopodia, and stress fibers, as well as increased actin polymerization ratio. Mentioned changes are related to YAP/TAZ nuclear localization, and induce cell spreading, adhesion, and invasion. Resistant cells also present much greater proteolytic activity, which is based on the increased expression and secretion of selected proteases, explaining their proinvasive phenotype. Understanding the molecular processes underlying resistance to BRAF and MEK targeted therapy is extremely important because it may lead to the discovery of new therapeutic targets for advanced melanoma.

## Supplementary Information

Supplementary Material 1.

Supplementary Material 2.

Supplementary Material 3.

Supplementary Material 4.

## Data Availability

The data underlying this article will be shared on reasonable request to the corresponding author.

## Abbreviations

BRAF: Serine/threonine-protein kinase B-raf
MAPK: Mitogen-activated protein kinase
MEK: Mitogen-activated protein kinase kinase
NRAS: N-ras proto-oncogene
ERK: Extracellular signal-regulated kinases
AKT: Protein kinase B
JNK: c-Jun N-terminal kinase
EGFR: Epidermal growth factor receptor
ErbB2: Receptor tyrosine-protein kinase erbB-2
MET: Hepatocyte growth factor receptor
PDGFRβ: Platelet-derived growth factor receptor beta
ECM: Extracellular matrix
RUNX2: RUNX family transcription factor 2
FA: Focal adhesions
FAK: Focal adhesion kinase
YAP: Yes-associated protein
TAZ: Transcriptional coactivator with PDZ-binding motif
MMPs: Matrix metalloproteinases
ADAM: Disintegrin and metalloproteinase domain-containing proteins
TIMPs: Tissue inhibitors of metalloproteinases
EMT: Epithelial-mesenchymal transition
SLUG: SNAI2; snail family transcriptional repressor 2
TGFβR: Transforming growth factor β receptors
SOX10: SRY-box transcription factor 10
ZEB1: Zinc finger E-box binding homeobox 1
Snail: SNAI1; snail family transcriptional repressor 1
CSC: Cancer stem cells
CD24: Signal transducer CD24
Nanog: Homeobox protein NANOG
ALCAM: Activated leukocyte cell adhesion molecule
