# Supplementary material for: Phenotype switching in highly invasive resistant to vemurafenib and cobimetinib melanoma cells
Source: Cell Commun Signal. 2025 Oct 21;23:449. doi: 10.1186/s12964-025-02452-0 (PMC12542628; doi:10.1186/s12964-025-02452-0)
Supplement: Supplementary file 1 — Supplementary Material 1. [file 12964_2025_2452_MOESM1_ESM.pdf]

WM9 PARVIN (Figure 3 panel C)

OTHER PROTEIN

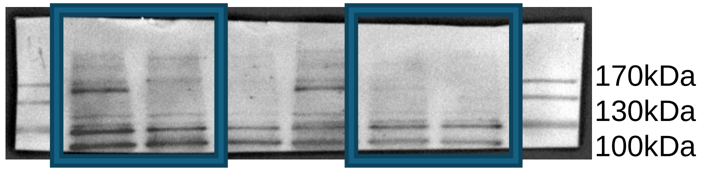

cut: 70kDa

PARVIN REPETITION 1 and 2

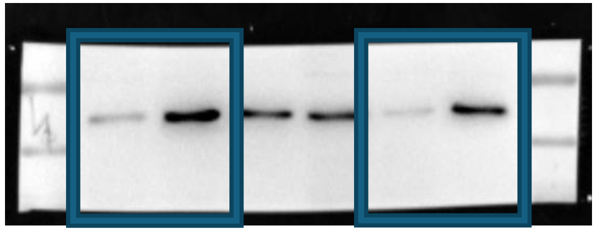

cut: 35kDa

OTHER PROTEIN

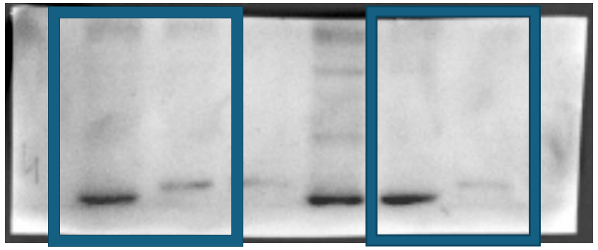

Ponceau S

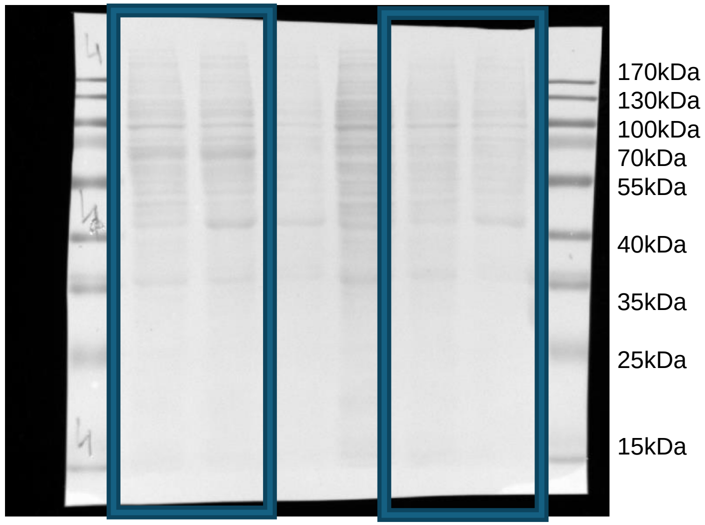

OTHER PROTEIN

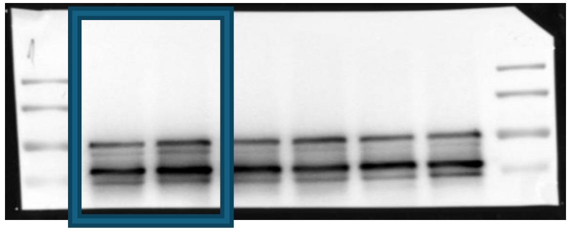

PARVIN REPETITION 3

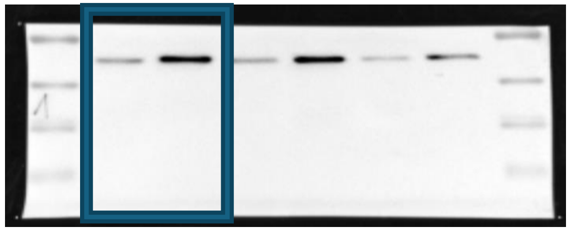

Ponceau S

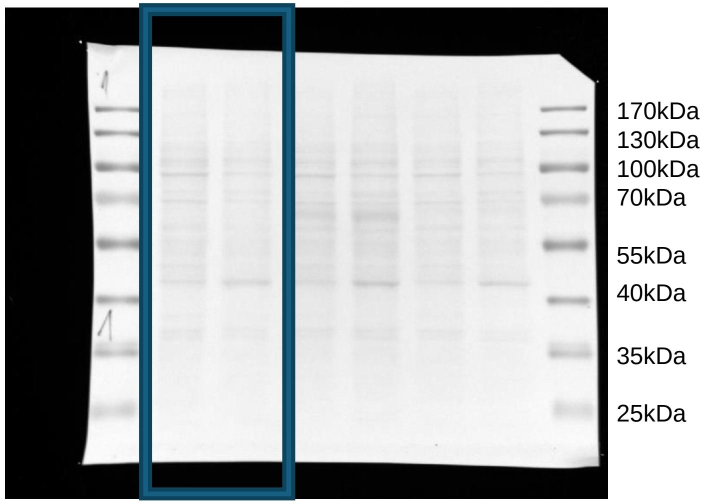

PARVIN Molecular Weight - **42 kDa**

CTRL- control **R**- resistant

Hs294T PARVIN (Figure 3 panel C)

OTHER PROTEIN

CTRL R CTRL R CTRL R

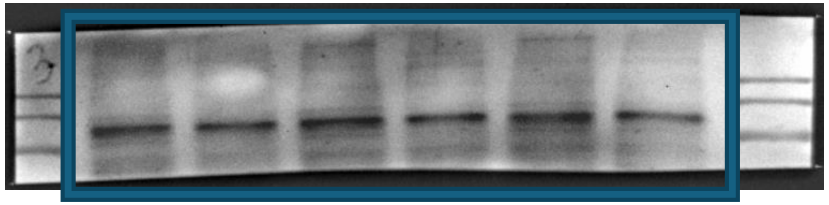

170kDa
130kDa
100kDa

cut: 70kDa

PARVIN REPETITION 1,2 and 3

CTRL R CTRL R CTRL R

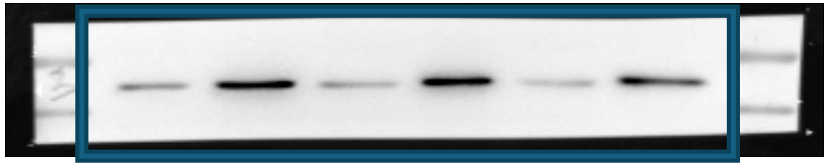

55kDa
40kDa

OTHER PROTEIN

CTRL R CTRL R CTRL R

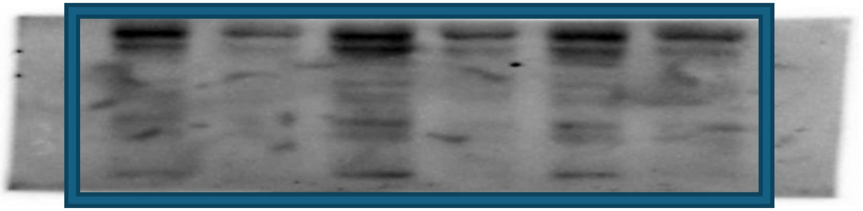

35kDa

Ponceau S

CTRL R CTRL R CTRL R

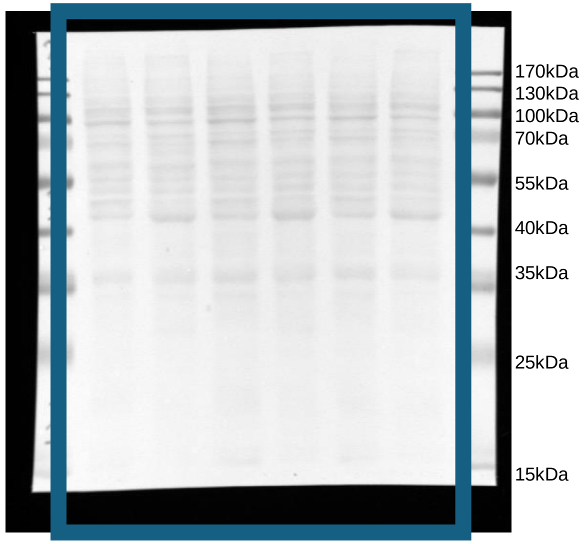

170kDa
130kDa
100kDa
70kDa
55kDa
40kDa
35kDa
25kDa
15kDa

PARVIN Molecular Weight - **42 kDa**

CTRL- control **R**- resistant

WM9 VINCULIN (Figure 3 panel D)

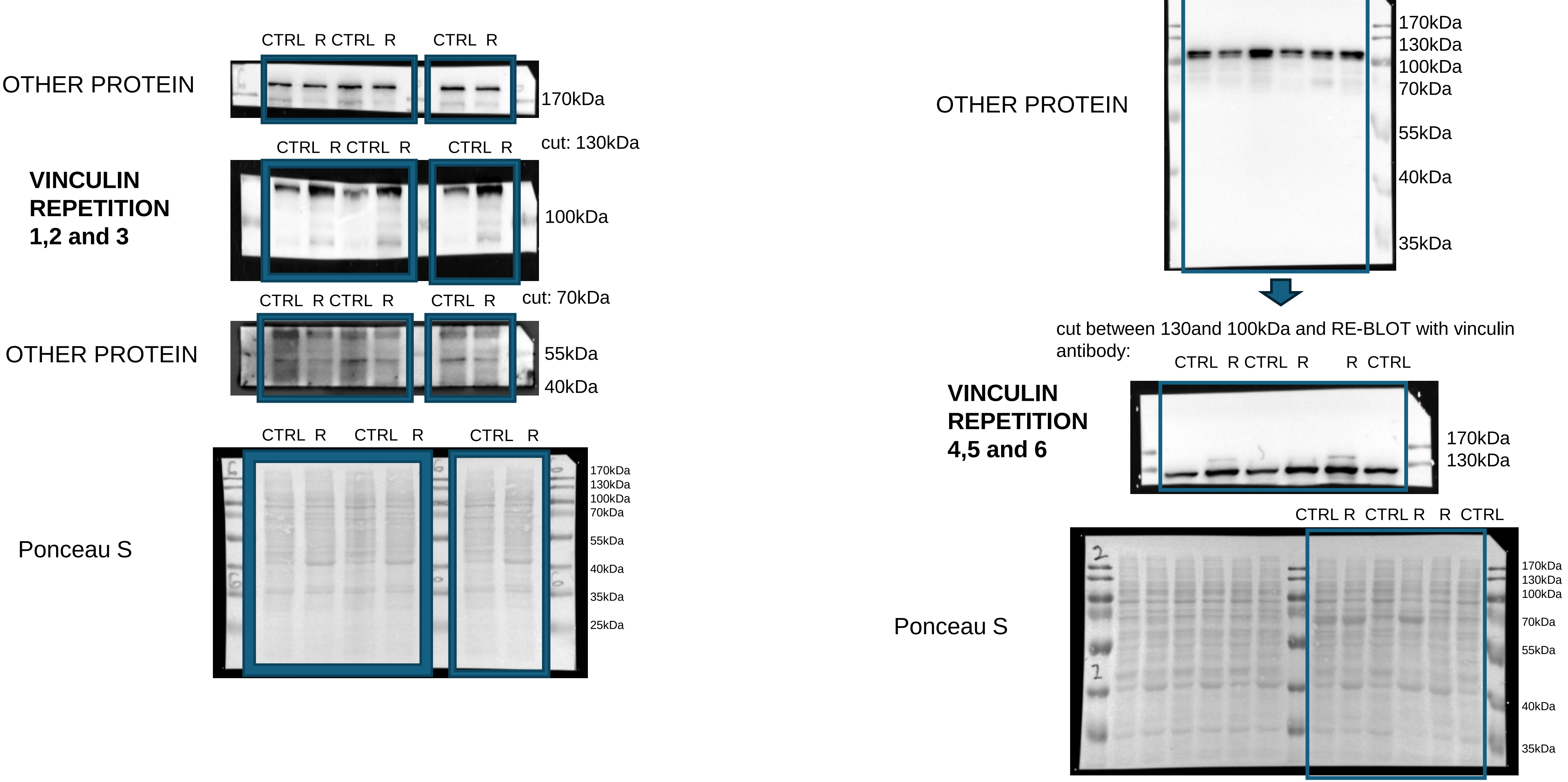

Vinculin Molecular Weight - **124 kDa** **CTRL**- control **R**- resistant

Hs294T VINCULIN (Figure 3 panel D)

VINCULIN REPETITION 1 and 2

OTHER PROTEIN

OTHER PROTEIN

Ponceau S

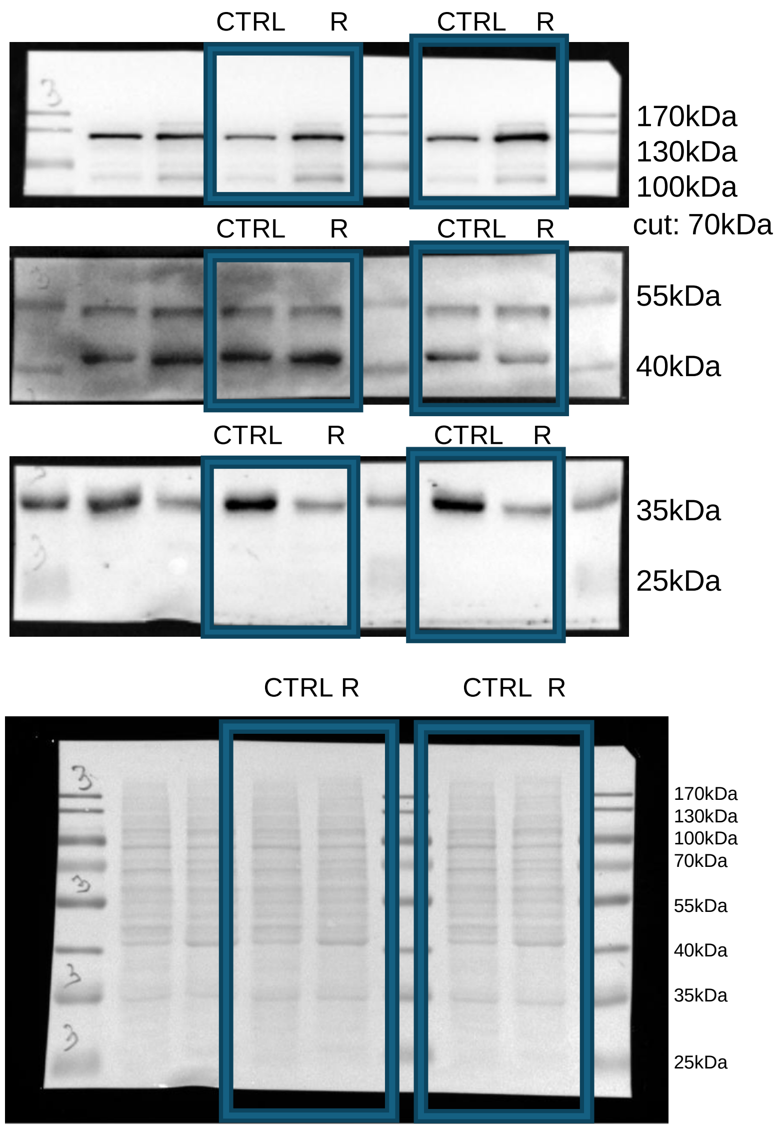

VINCULIN REPETITION 3,4 and 5

OTHER PROTEIN

OTHER PROTEIN

Ponceau S

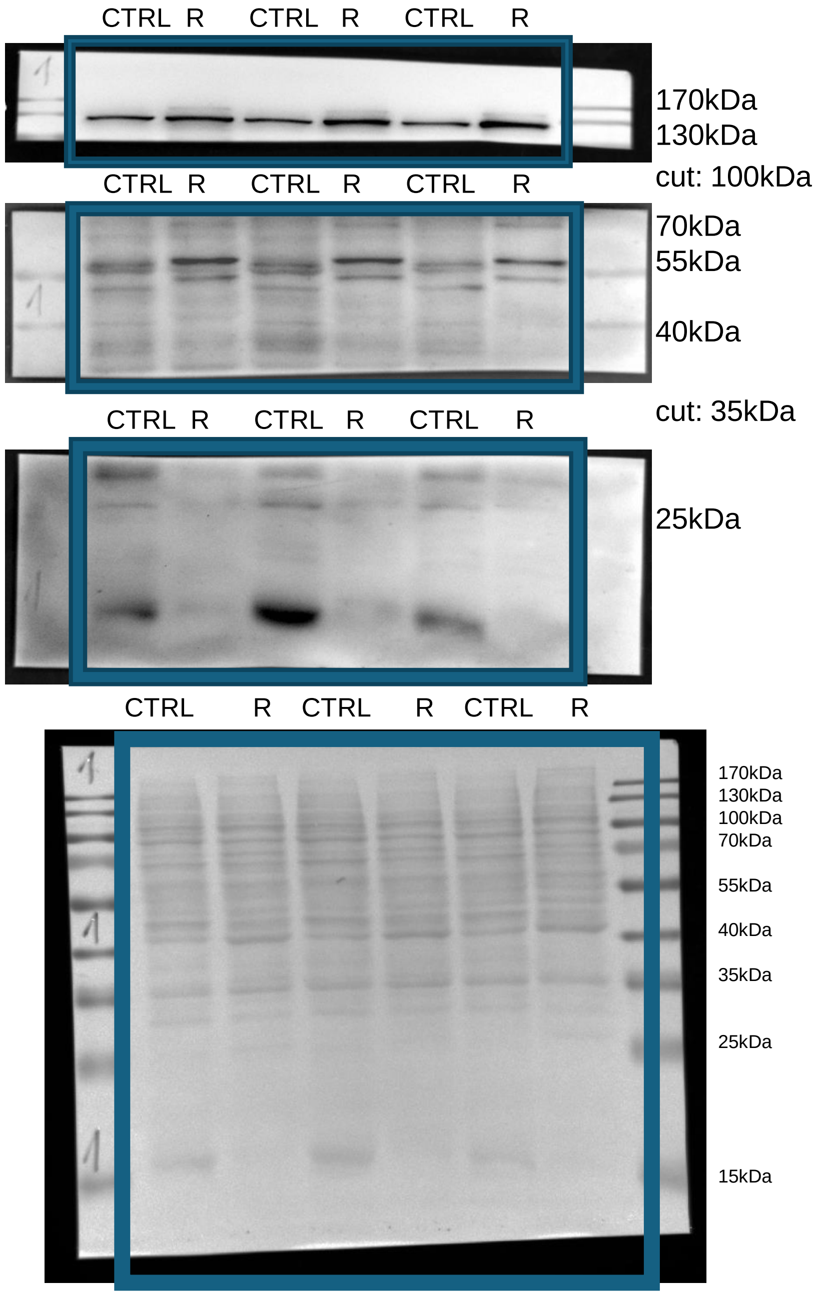

Vinculin Molecular Weight - **124 kDa**

CTRL- control **R**- resistant

WM9 p-FAK (Figure 3 panel E)

p-FAK REPETITION 1,2 and 3

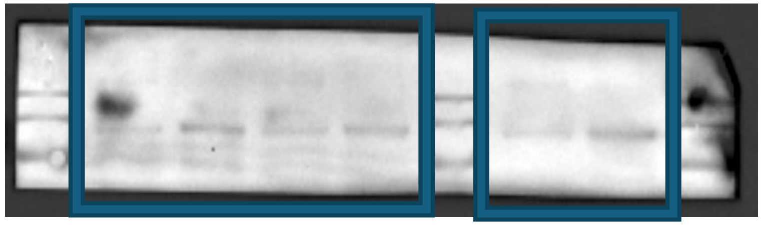

OTHER PROTEIN

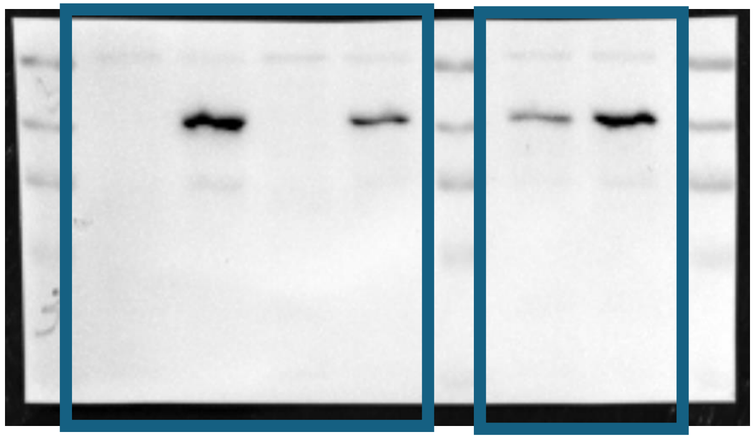

Ponceau S

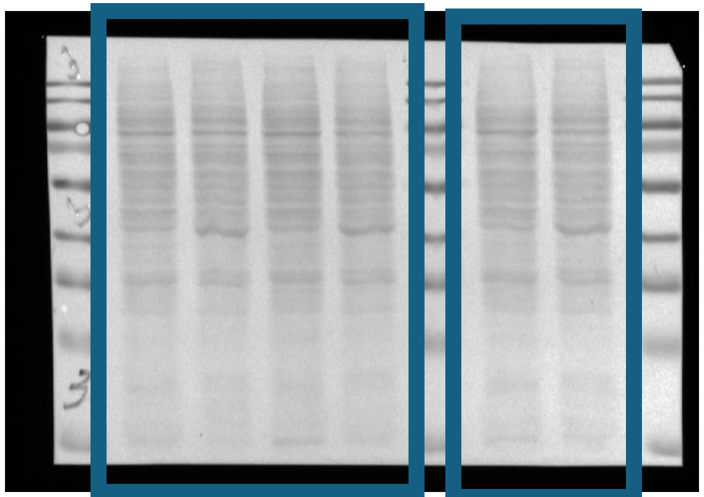

pFAK Molecular Weight - **119 kDa**

CTRL- control **R**- resistant

WM9 FAK (Figure 3 panel E)

FAK REPETITION 1,2 and 3

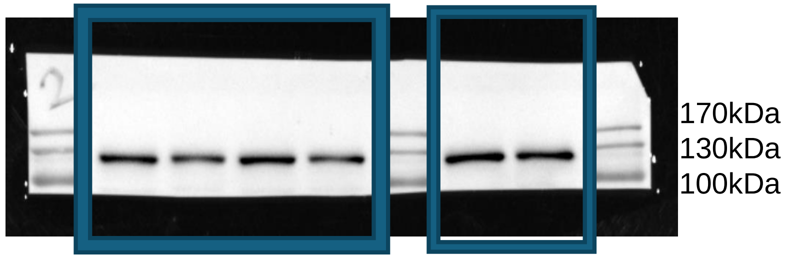

OTHER PROTEIN

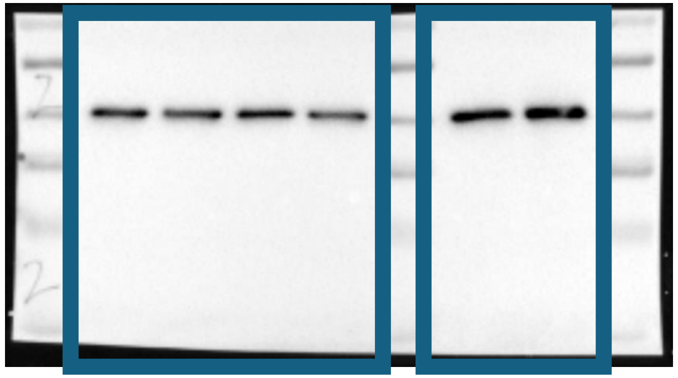

Ponceau S

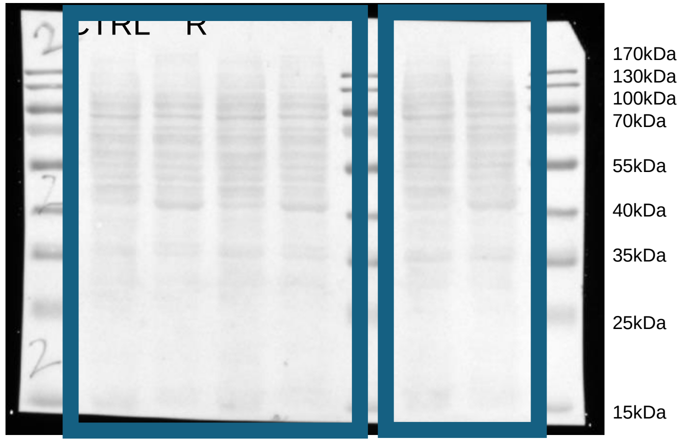

FAK Molecular Weight - **119 kDa**

CTRL- control **R**- resistant

Hs294T p-FAK (Figure 3 panel E)

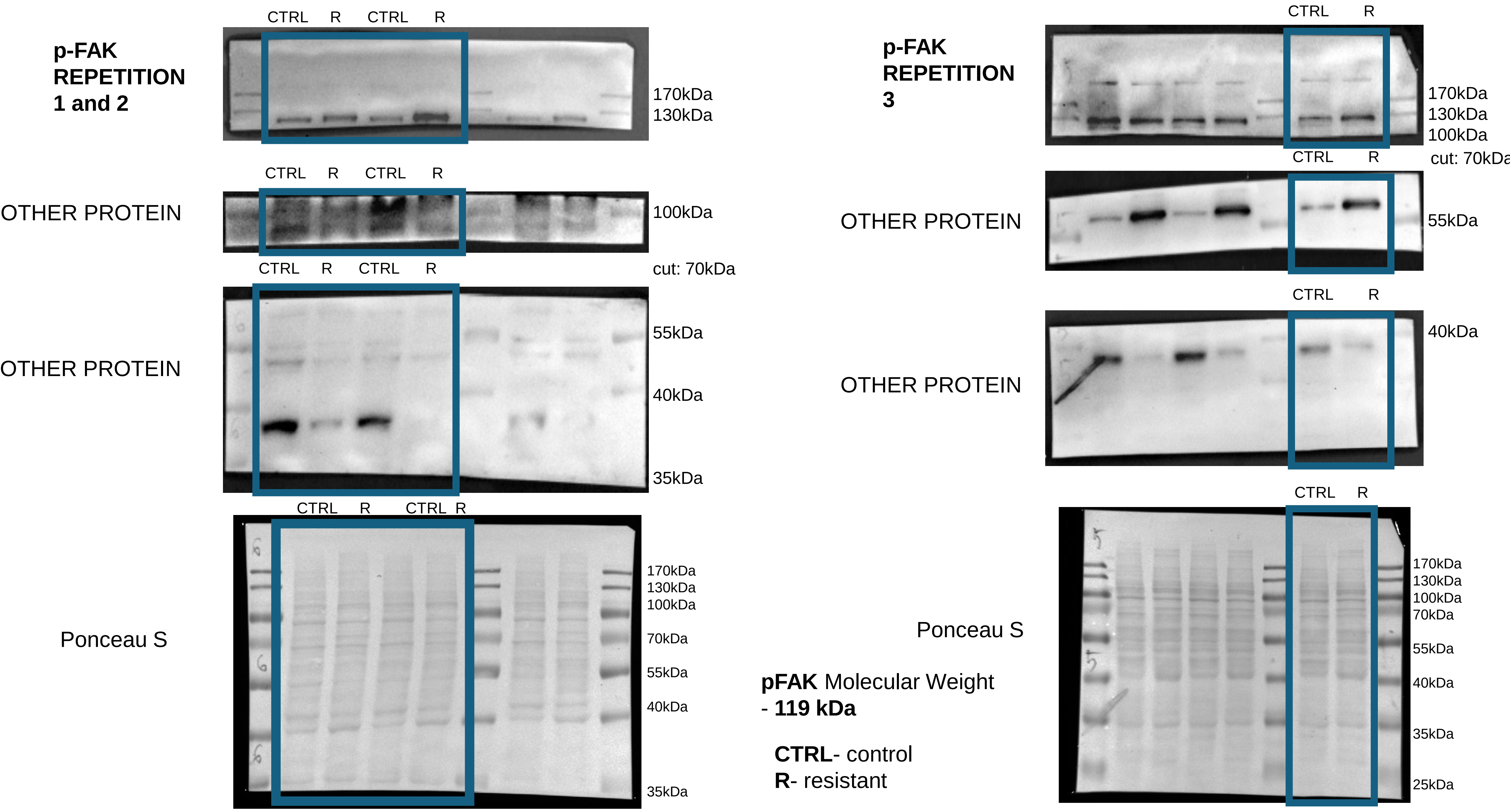

pFAK Molecular Weight - **119 kDa**

CTRL- control
R- resistant

Hs294T FAK (Figure 3 panel E)

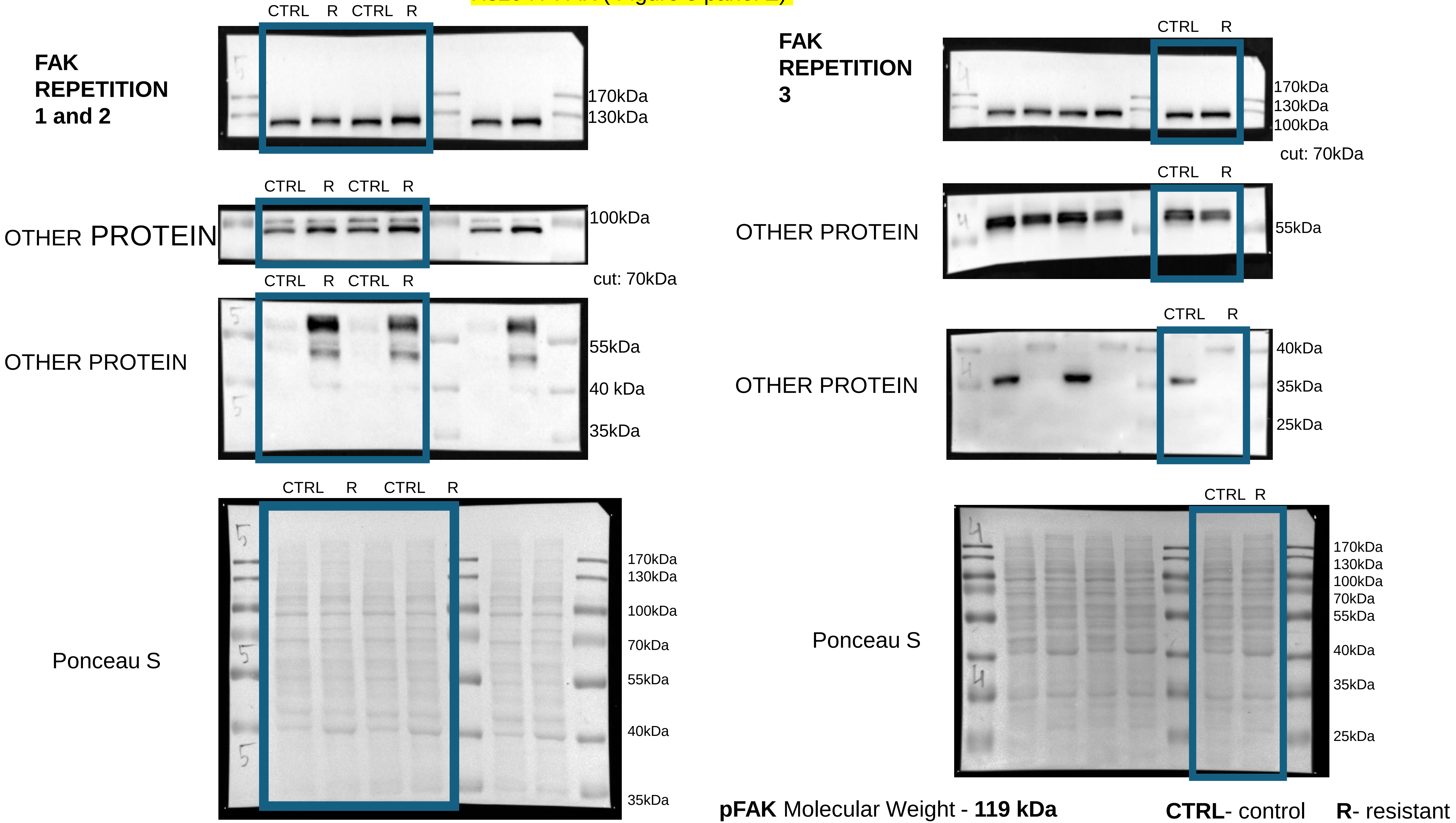

pFAK Molecular Weight - **119 kDa** **CTRL**- control **R**- resistant

WM9 RUNX2 (Figure 1 panel C)

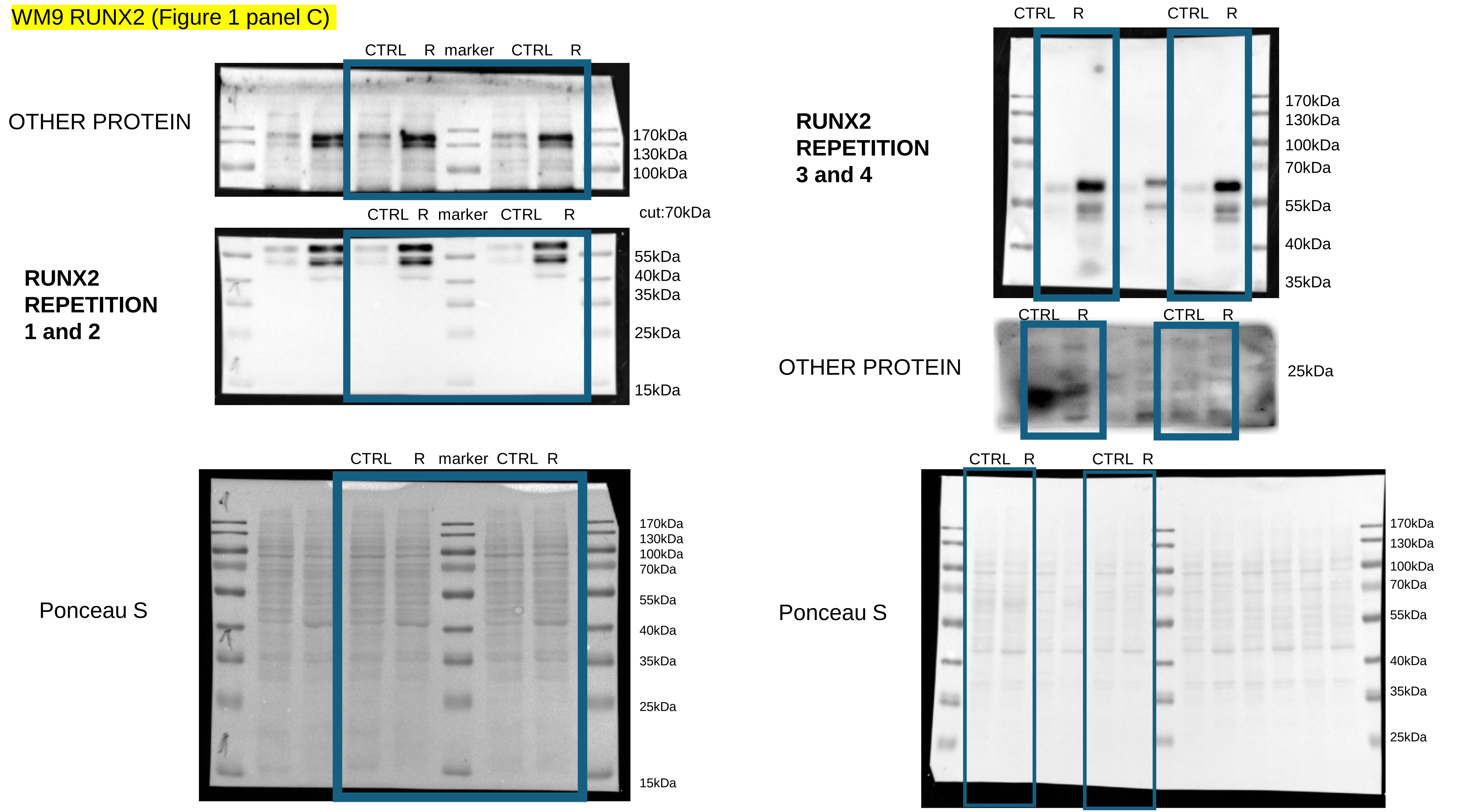

RUNX2 Molecular Weight - **57 kDa** **CTRL**- control **R**- resistant

Hs294T RUNX2 (Figure 1 panel C)

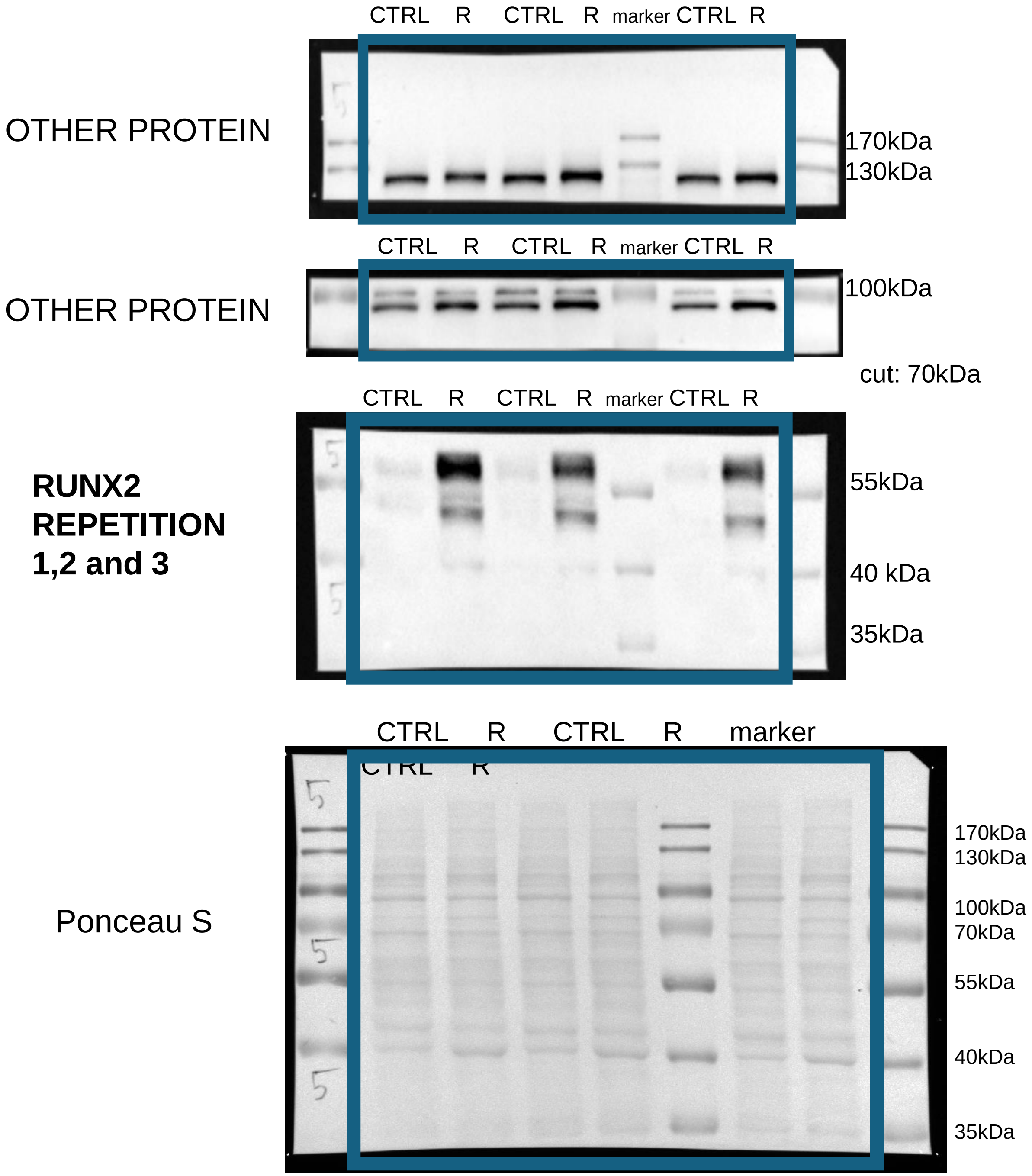

RUNX2 Molecular Weight - **57 kDa**

CTRL- control **R**- resistant

WM9 β-ACTIN (Figure 5 panel A)

OTHER PROTEIN

CTRL R CTRL R

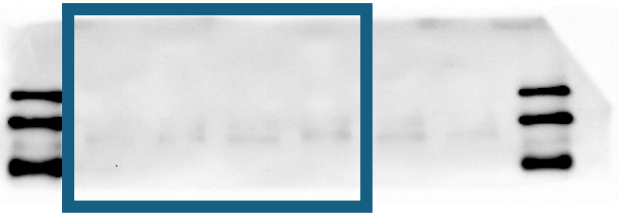

170kDa
130kDa
100kDa

OTHER PROTEIN

CTRL R CTRL R

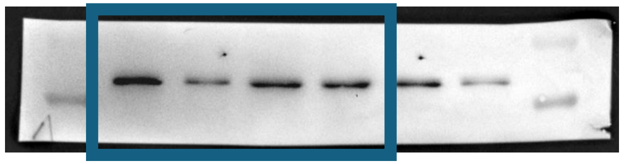

70kDa
55kDa

β-ACTIN REPETITION 1 and 2

CTRL R CTRL R

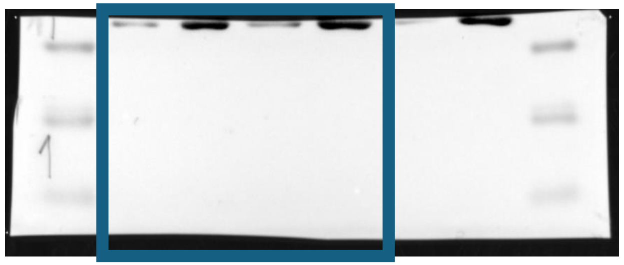

40kDa
35kDa
25kDa

Ponceau S

CTRL R CTRL R

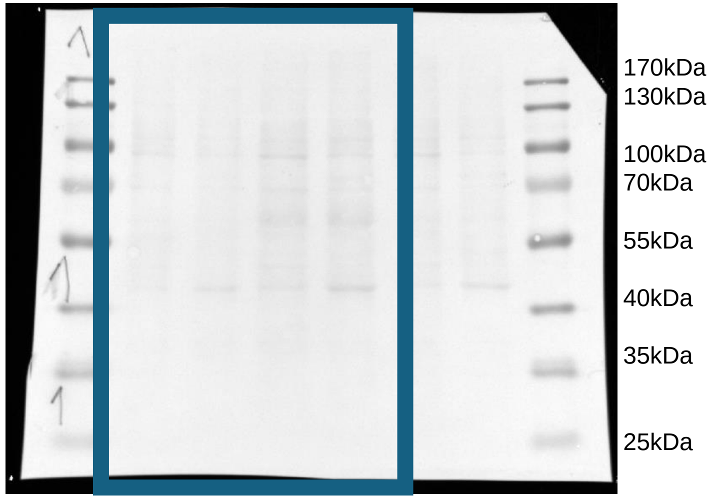

170kDa
130kDa
100kDa
70kDa
55kDa
40kDa
35kDa
25kDa

Nothing above 100kDa visualised

β-ACTIN REPETITION 3, 4 and 5

CTRL R CTRL R CTRL R

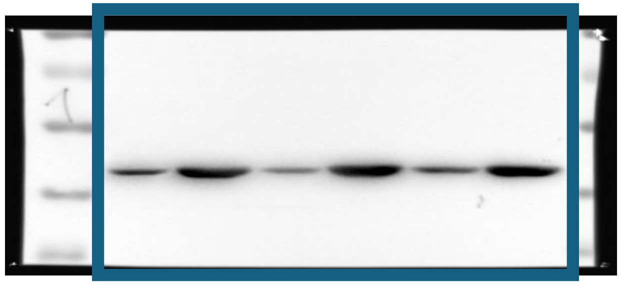

100kDa
70kDa
55kDa
40kDa
35kDa

CTRL R CTRL R CTRL R

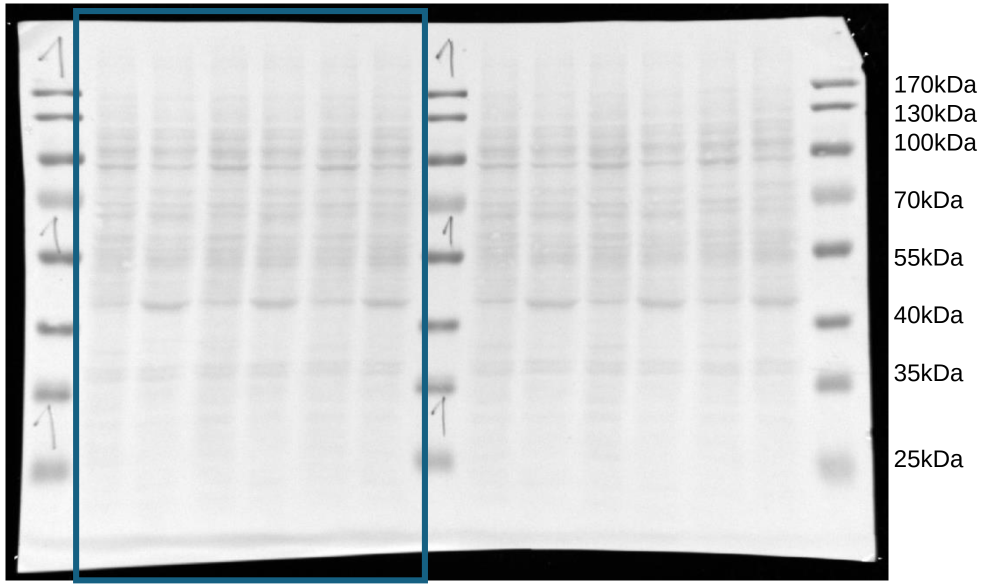

170kDa
130kDa
100kDa
70kDa
55kDa
40kDa
35kDa
25kDa

β actin Molecular Weight - **42 kDa**

CTRL- control **R**- resistant

Hs294T β-ACTIN (Figure 5 panel A)

β actin Molecular Weight - **42 kDa**

OTHER PROTEIN

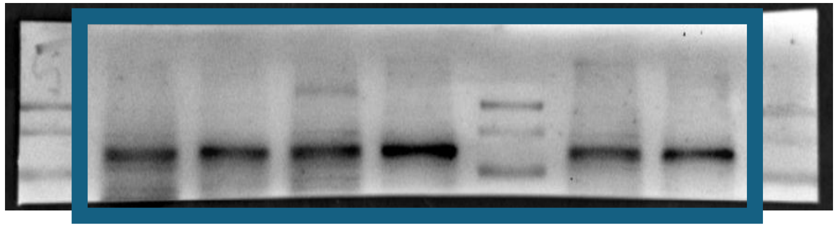

OTHER PROTEIN

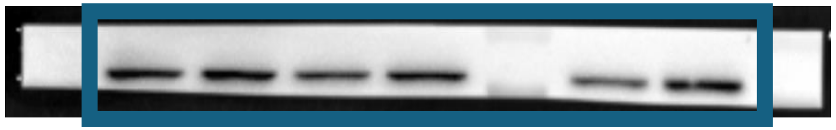

β-ACTIN REPETITION 1, 2 and 3

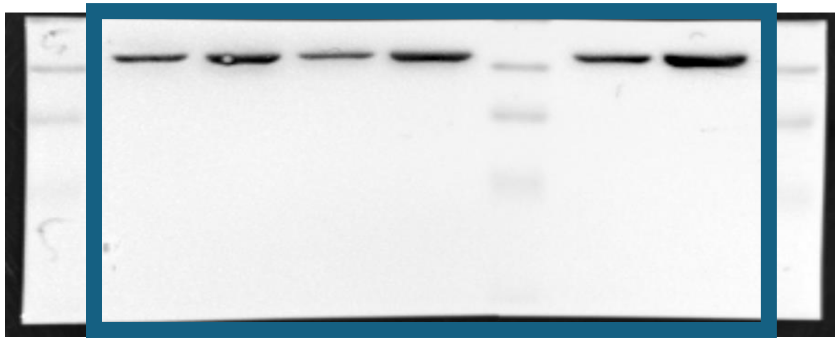

Ponceau S

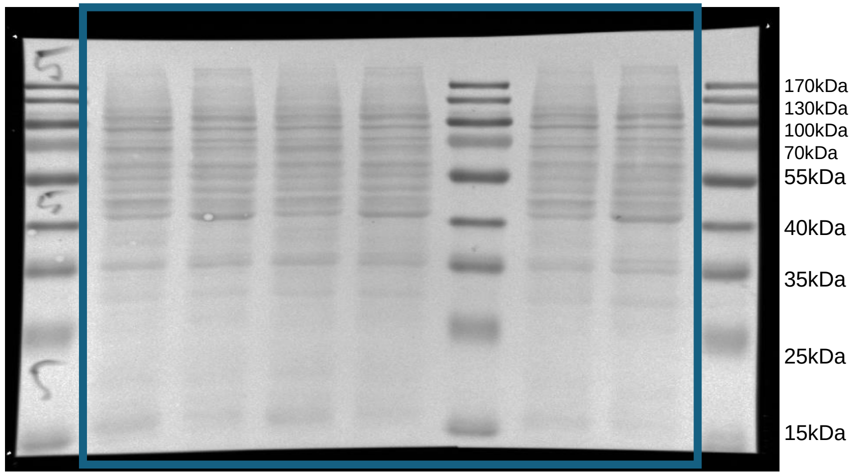

OTHER PROTEIN

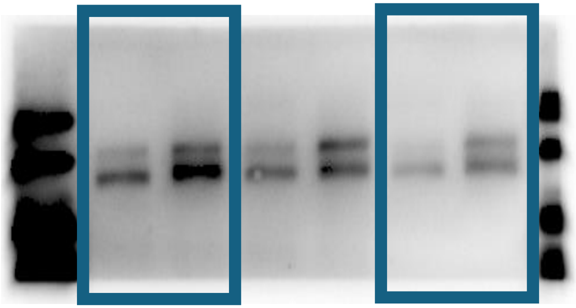

β-ACTIN REPETITION 4 and 5

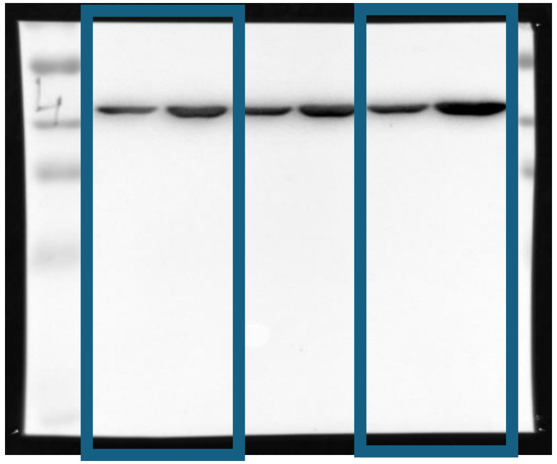

Ponceau S

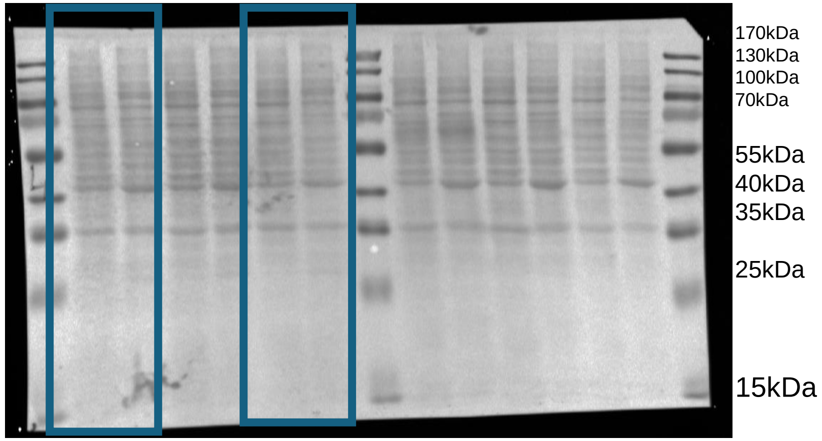

CTRL- control
R- resistant

WM9 γ-ACTIN (Figure 5 panel B)

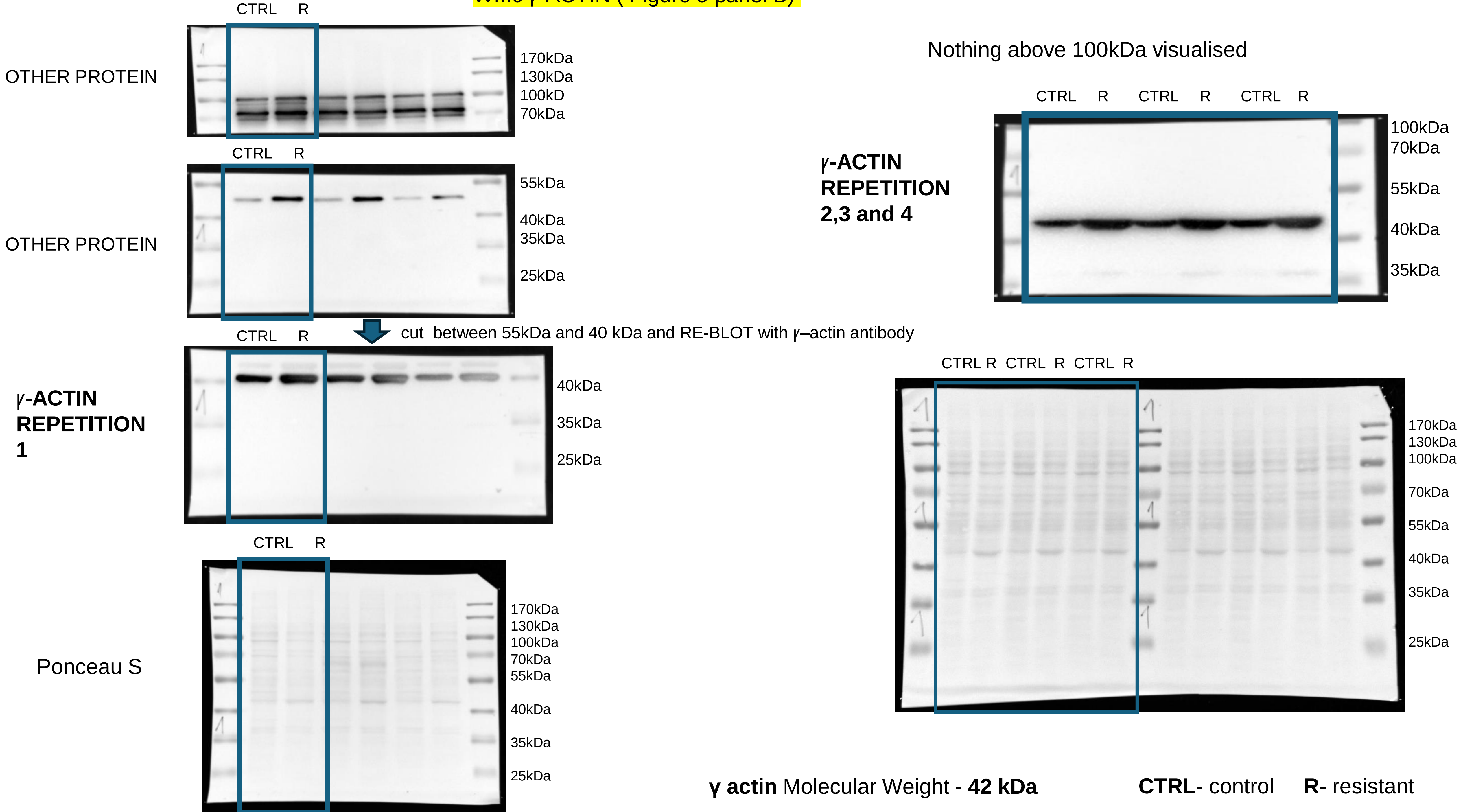

γ actin Molecular Weight - **42 kDa** **CTRL**- control **R**- resistant

Hs294T γ-ACTIN (Figure 5 panel B)

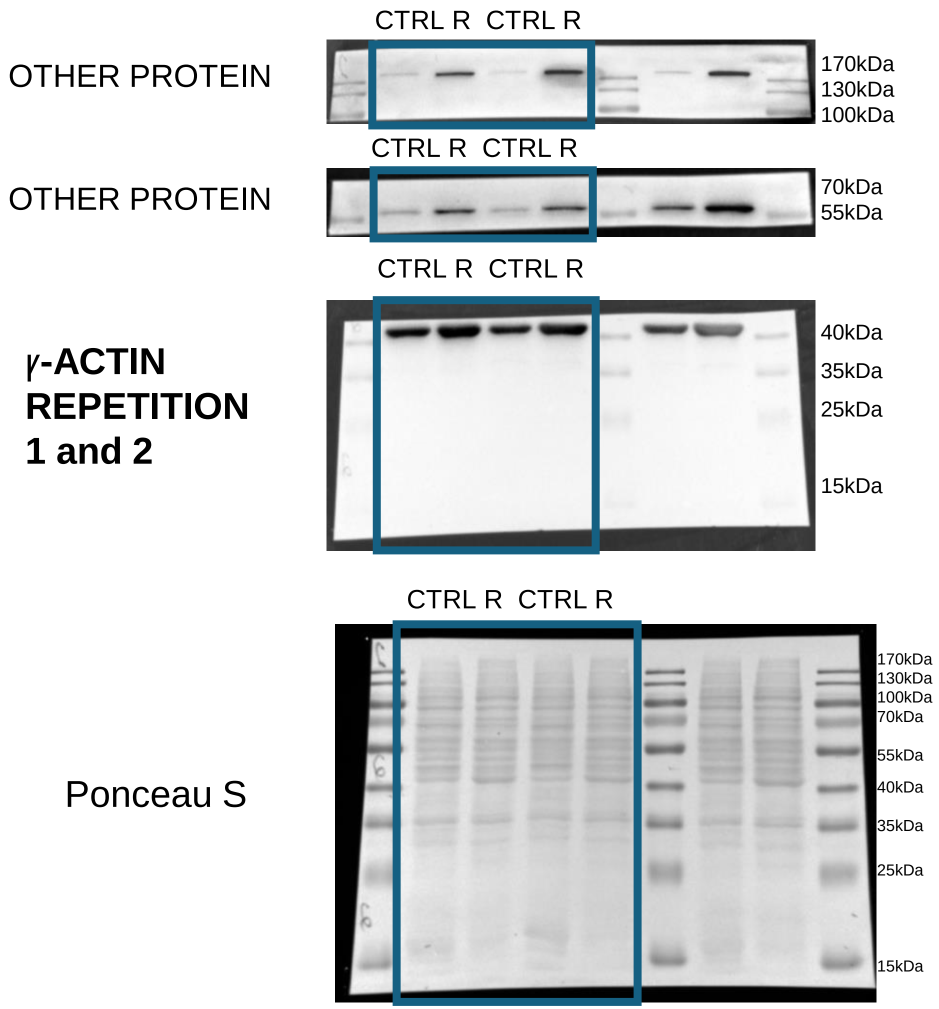

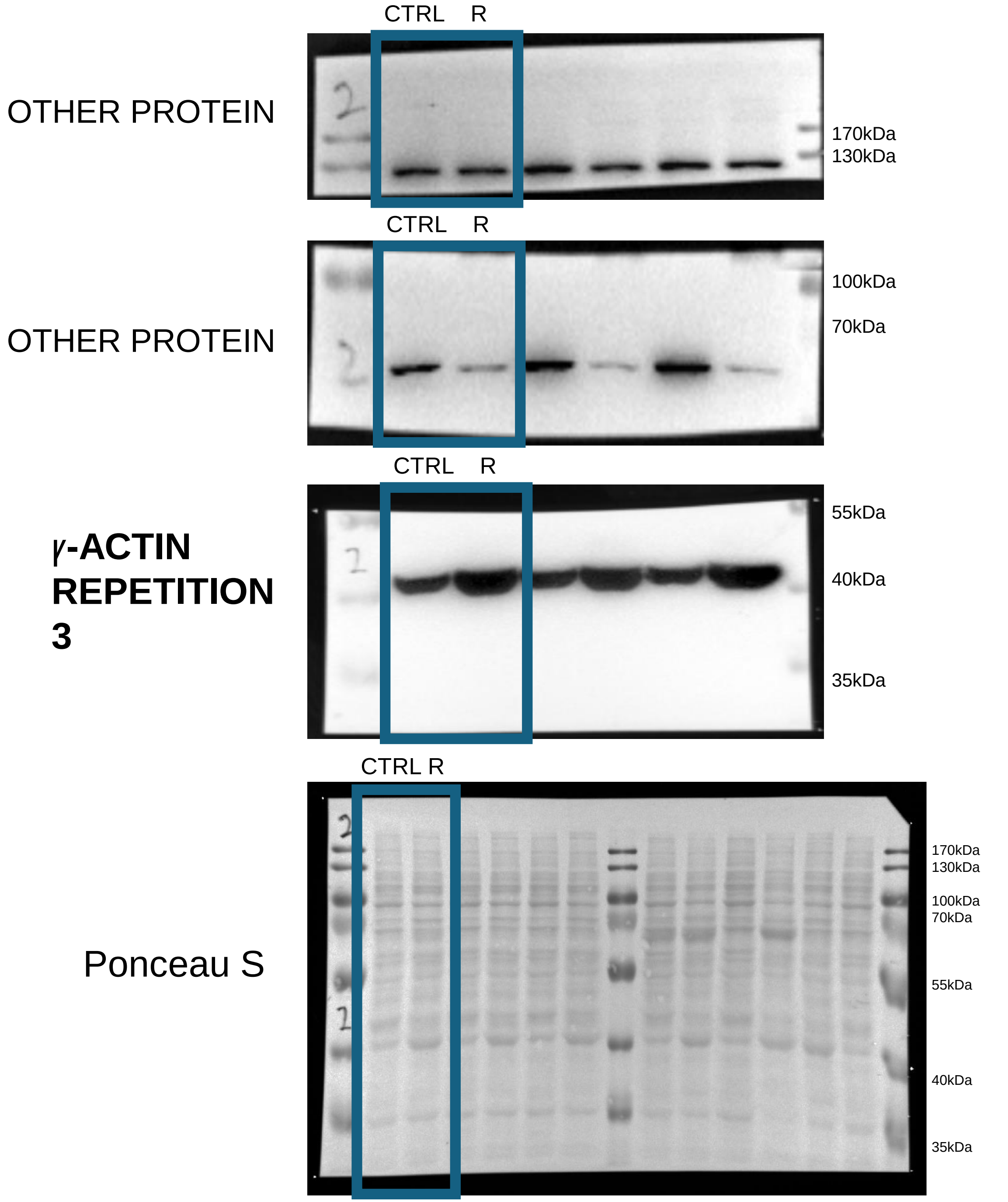

γ actin Molecular Weight - **42 kDa** **CTRL**- control **R**- resistant

WM9 MMP-2 (Figure 7 panel A)

MMP-2 REPETITION 1,2 and 3

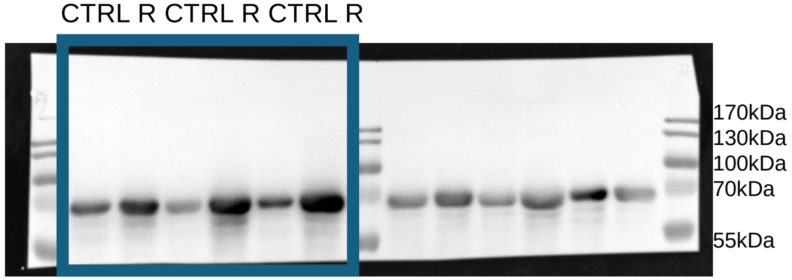

OTHER PROTEIN

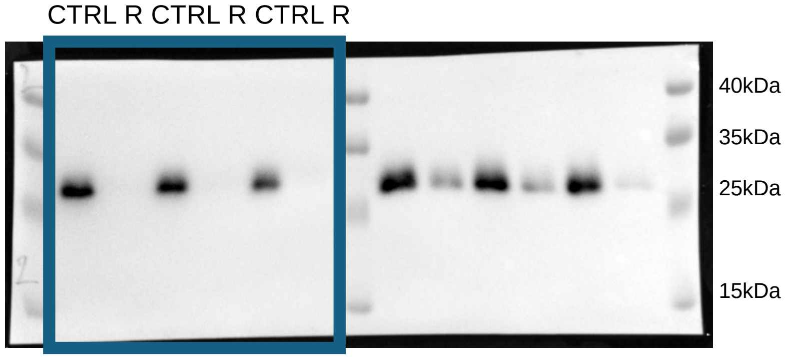

Ponceau S

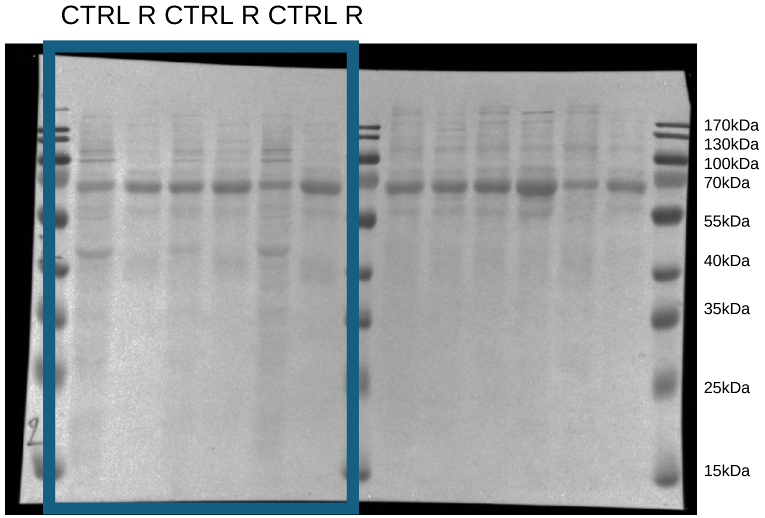

MMP-2 REPETITION 4

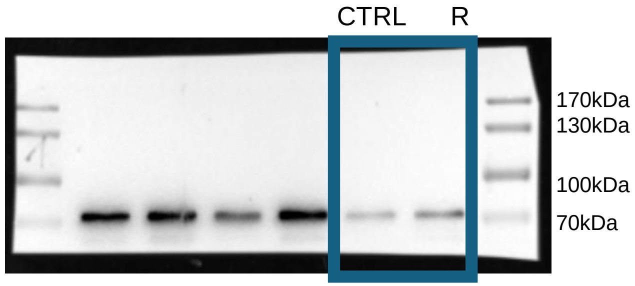

OTHER PROTEIN

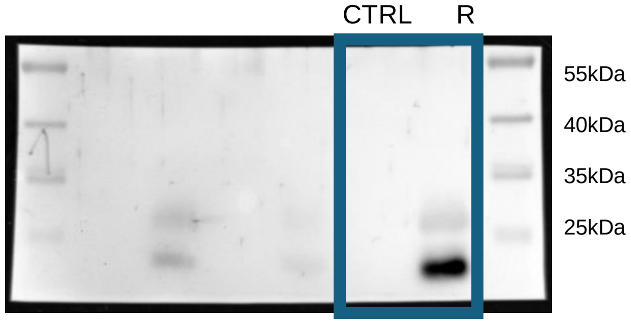

Ponceau S

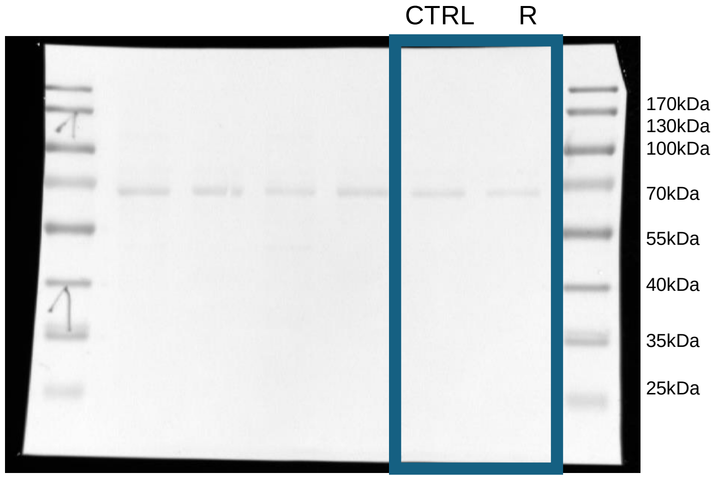

MMP2 Molecular Weight - **72 kDa** **CTRL**- control **R**- resistant

Hs294T MMP-2 (Figure 7 panel A)

MMP-2 REPETITION 1 and 2

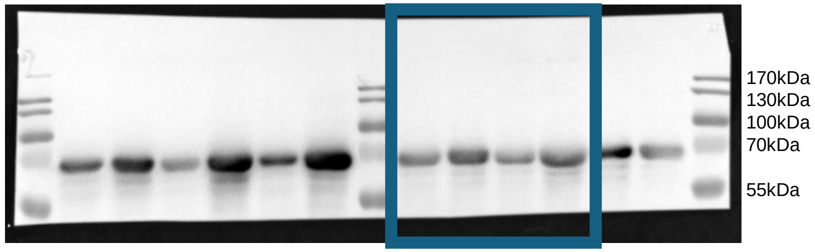

OTHER PROTEIN

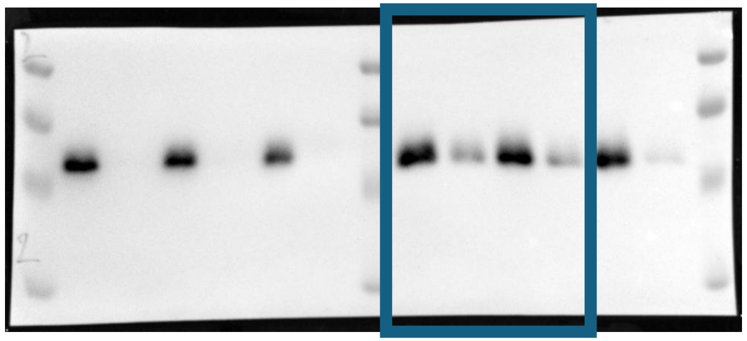

Ponceau S

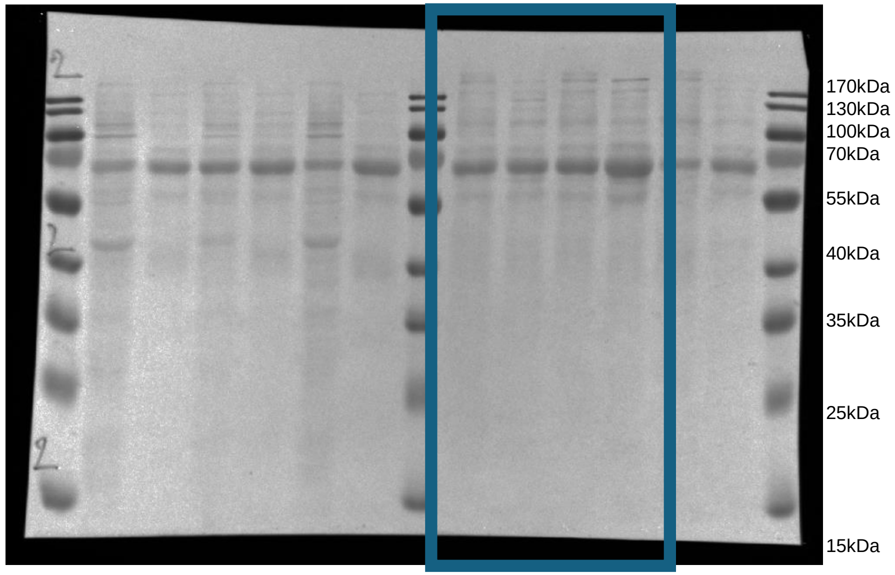

MMP-2 REPETITION 3 and 4

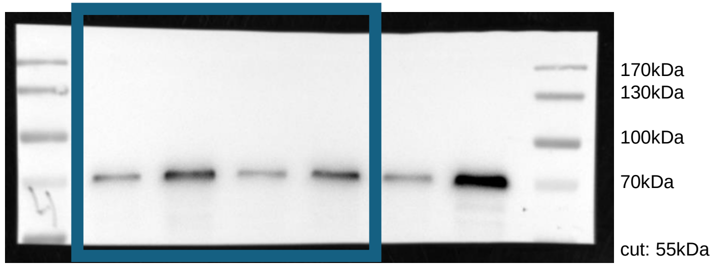

Nothing below 55kDa visualised

Ponceau S

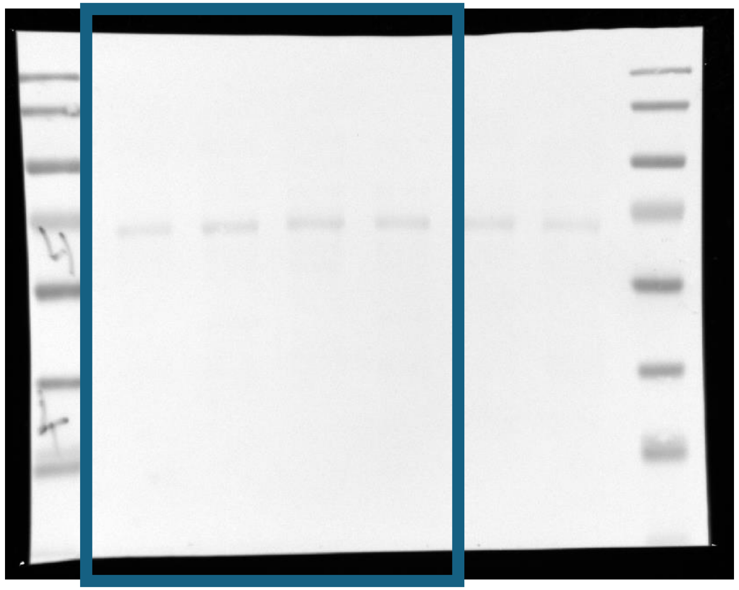

MMP2 Molecular Weight - **72 kDa**

CTRL- control **R**- resistant

WM9 MMP-9 (Figure 7 panel B)

MMP-9 REPETITION 1 and 2

CTRL R CTRL R

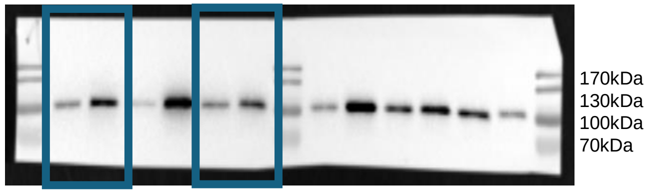

170kDa
130kDa
100kDa
70kDa

OTHER PROTEIN

CTRL R CTRL R

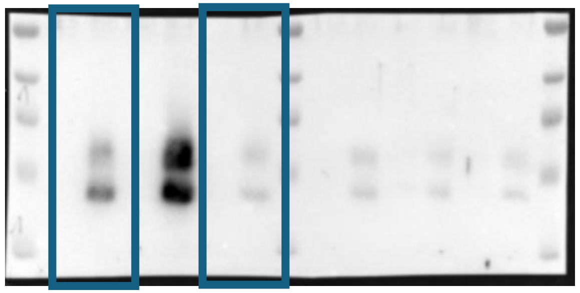

55kDa
40kDa
35kDa
25kDa
15kDa

Ponceau S

CTRL R CTRL R

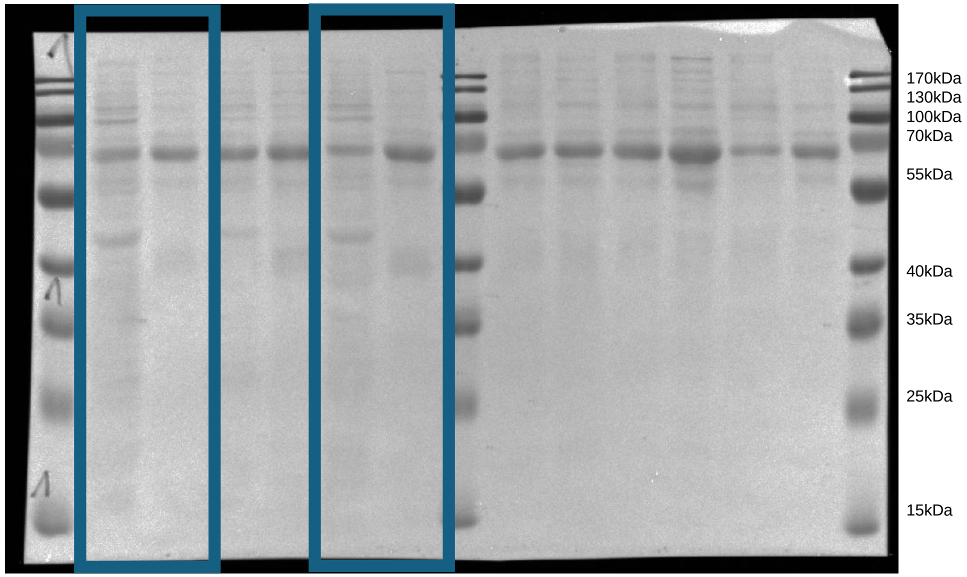

170kDa
130kDa
100kDa
70kDa
55kDa
40kDa
35kDa
25kDa
15kDa

MMP-9 REPETITION 3 and 4

CTRL R CTRL R

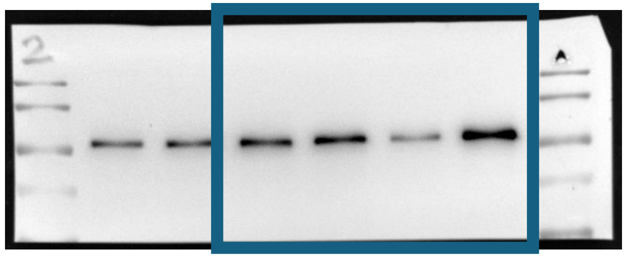

170kDa
130kDa
100kDa
70kDa
55kDa

OTHER PROTEIN

CTRL R CTRL R

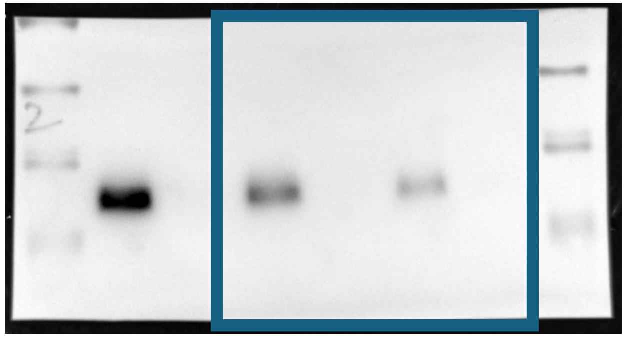

40kDa
35kDa
25kDa

Ponceau S

CTRL R CTRL R

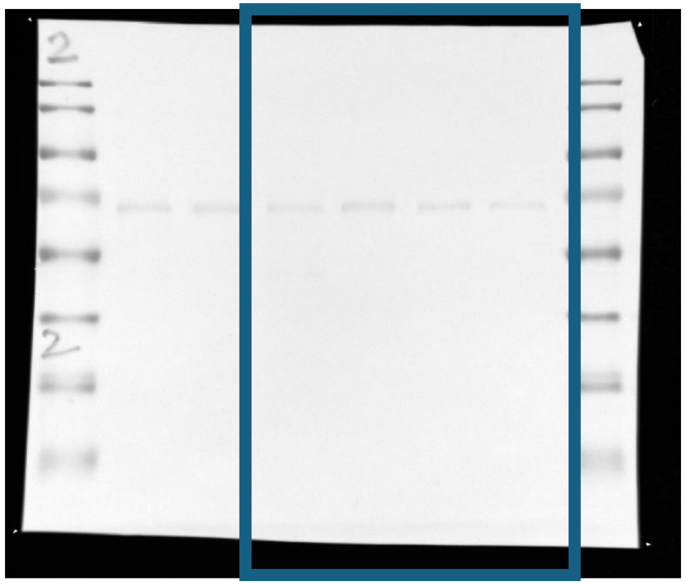

170kDa
130kDa
100kDa
70kDa
55kDa
40kDa
35kDa
25kDa

MMP9 Molecular Weight - **92 kDa**

CTRL- control **R**- resistant

Hs294T MMP-9 (Figure 7 panel B)

MMP-9 REPETITION 1

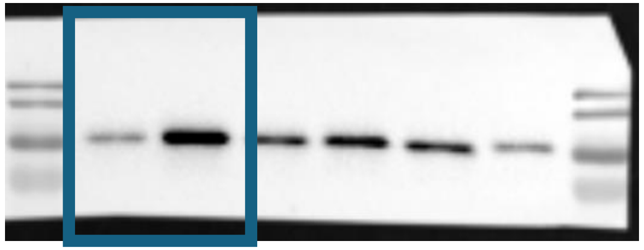

OTHER PROTEIN

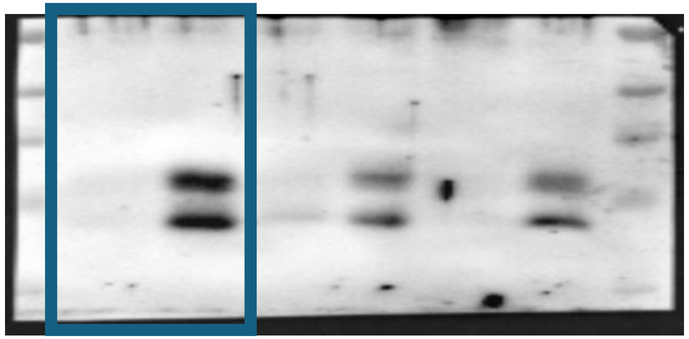

Ponceau S

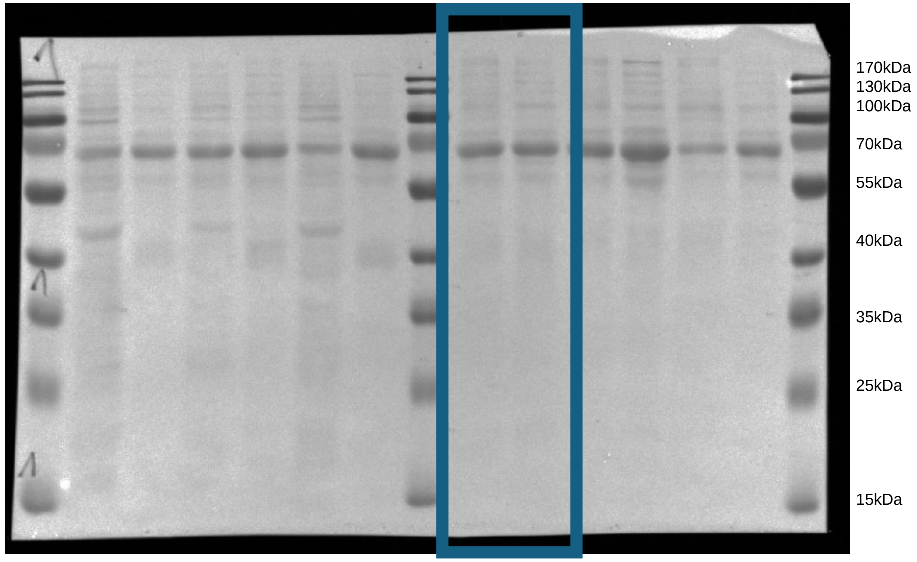

MMP-9 REPETITION 2 and 3

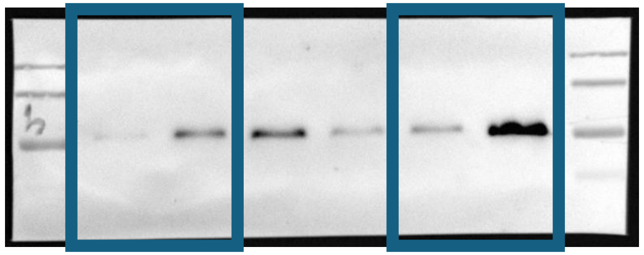

OTHER PROTEIN

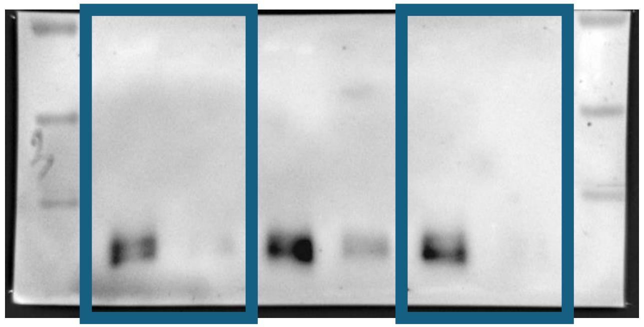

Ponceau S

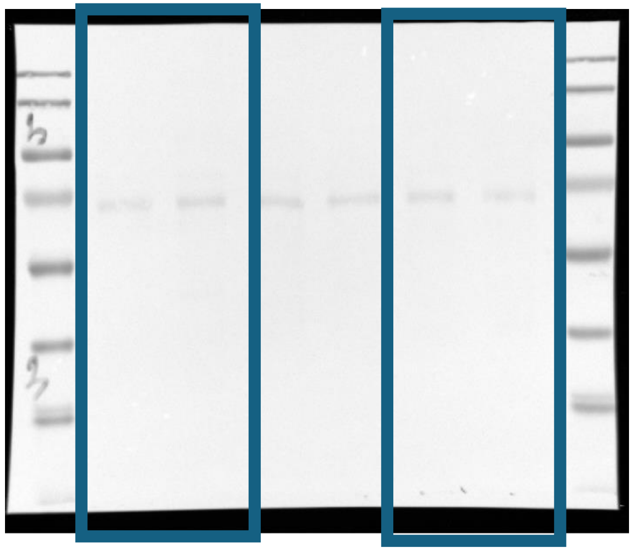

MMP9 Molecular Weight - **92 kDa** **CTRL**- control **R**- resistant

WM9 and Hs294T MMP-2 (Figure 7 panel C)

Gelatin zymography

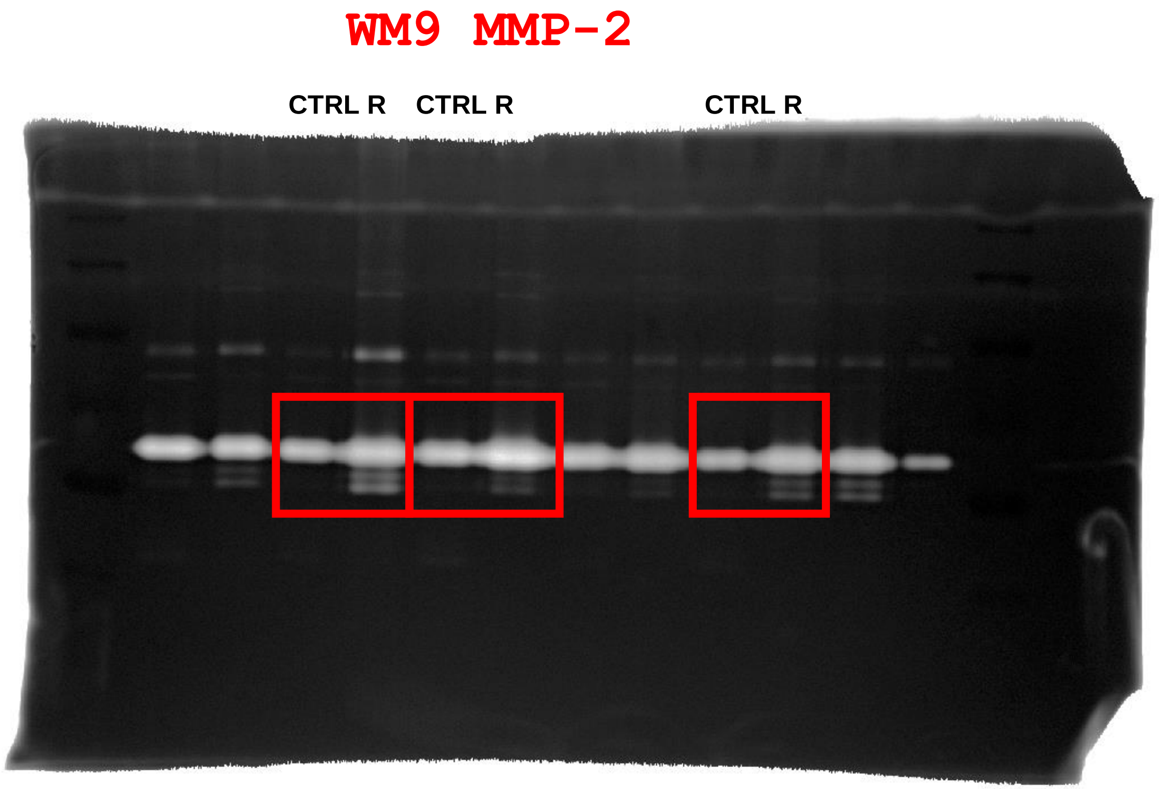

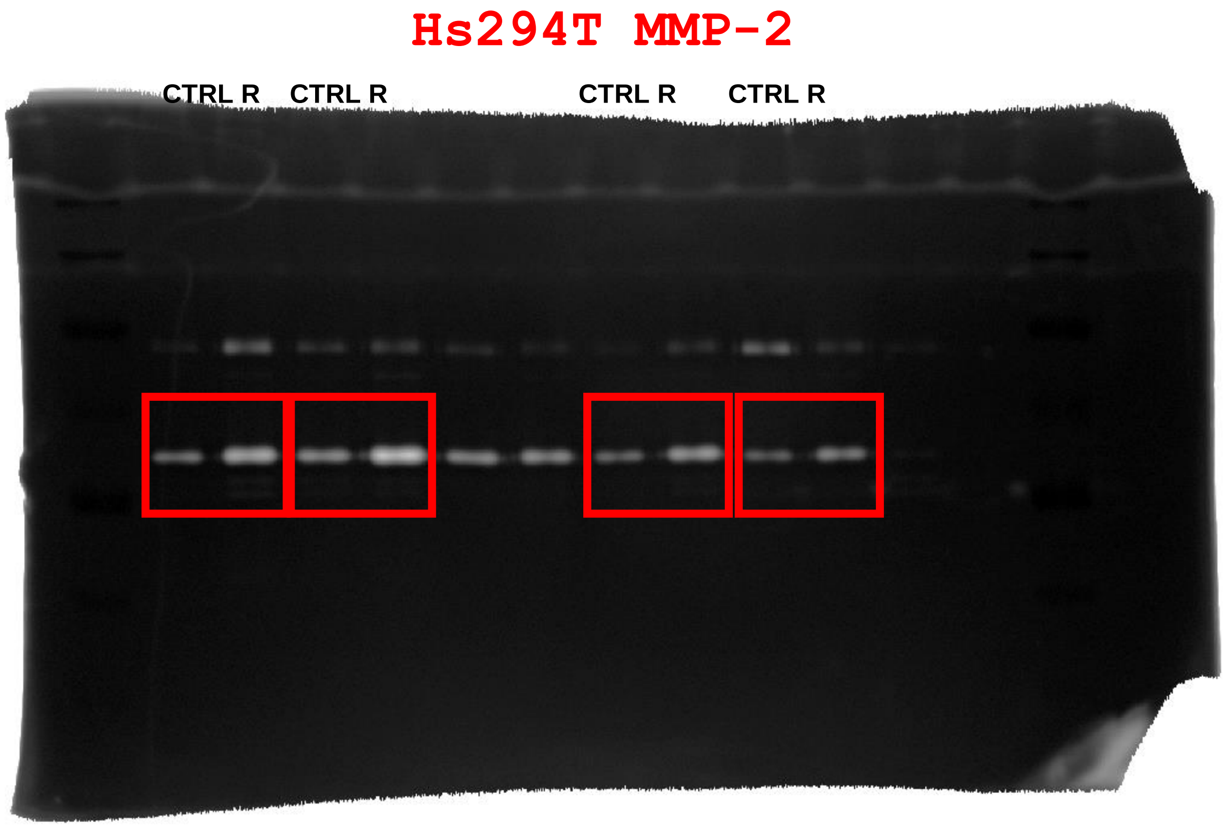

MMP2 Molecular Weight - **72 kDa**

CTRL- control **R**- resistant

WM9 and Hs294T MMP-9 (Figure 7 panel D)

Gelatin zymography

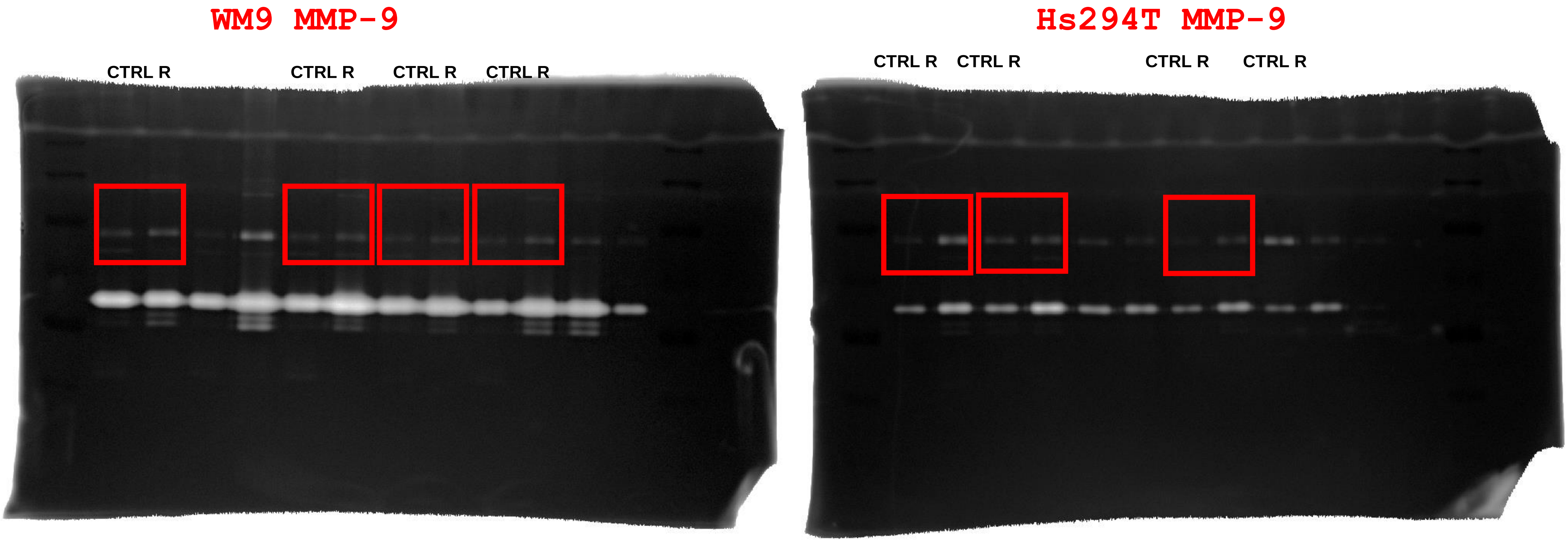

MMP9 Molecular Weight - **92 kDa**

CTRL- control **R**- resistant
