# Supplementary figures and images for: Phenotype switching in highly invasive resistant to vemurafenib and cobimetinib melanoma cells
Source: Cell Commun Signal. 2025 Oct 21;23:449. doi: 10.1186/s12964-025-02452-0 (PMC12542628; doi:10.1186/s12964-025-02452-0)

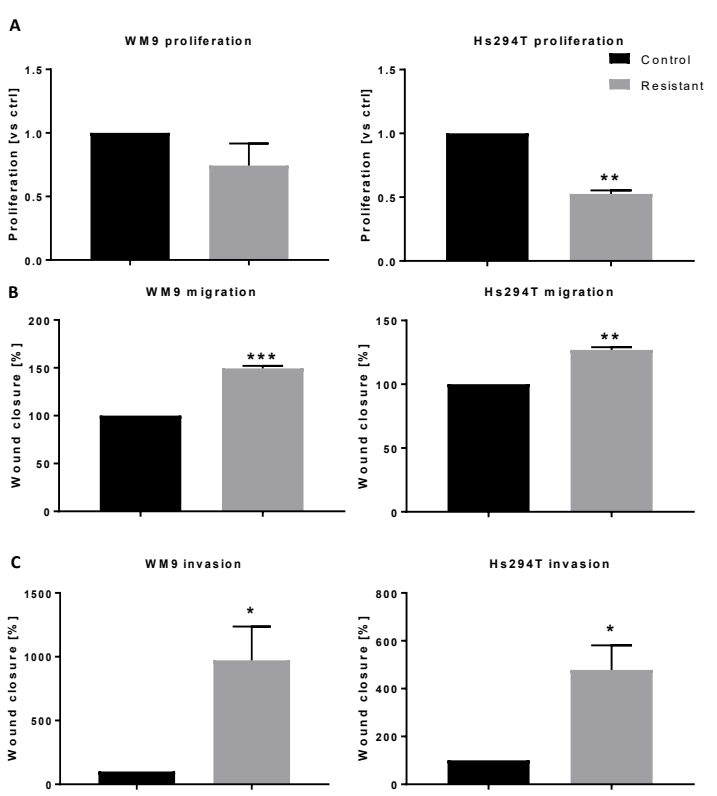

Supplement: Supplementary file 2 — Supplementary Material 2. [file 12964_2025_2452_MOESM2_ESM.tif]

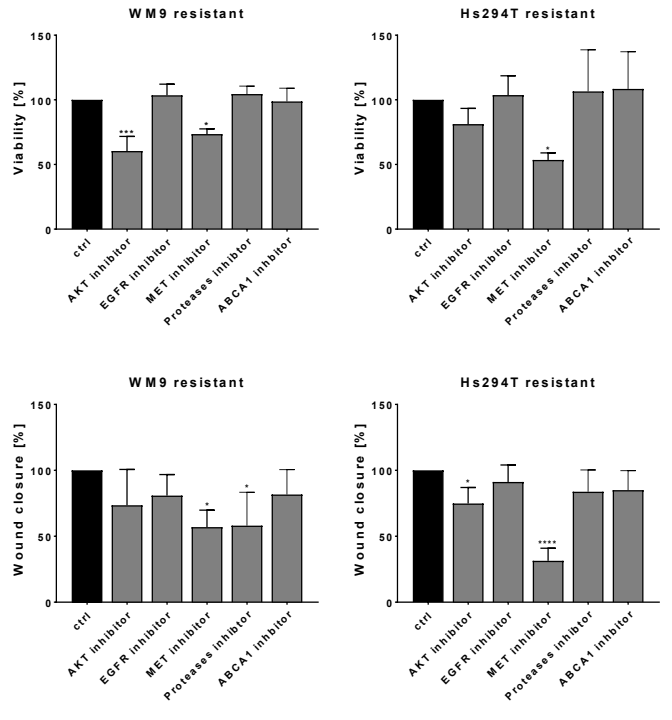

Supplement: Supplementary file 3 — Supplementary Material 3. [file 12964_2025_2452_MOESM3_ESM.tif]
