# Supplementary material for: Phenotype switching in highly invasive resistant to vemurafenib and cobimetinib melanoma cells
Source: Cell Commun Signal. 2025 Oct 21;23:449. doi: 10.1186/s12964-025-02452-0 (PMC12542628; doi:10.1186/s12964-025-02452-0)
Supplement: Supplementary file 4 — Supplementary Material 4. [file 12964_2025_2452_MOESM4_ESM.docx]

**Supplementary Figures:**

**Supplemental Figure 1** Viability, migration, and invasion of control and resistant melanoma cells after BRAF/MEK inhibitors withdrawal. After a few weeks of culturing cells in medium devoid of 0,01% DMSO (control cells) or 0,4µM vemurafenib/ 0,4µM cobimetinib (resistant cells), their proliferation (A), migration (B), and invasion (C) were examined. Statistical significance was defined as p ≤ 0.05 (*), p ≤ 0.01 (**), and p ≤ 0.001 (***).

**Supplemental Figure 2** Viability and invasion of resistant melanoma cells after treatment with selected inhibitors. An XTT test was used to analyze cell viability. A scratch wound assay, in which cells migrate to close the wound, was used to evaluate the rate of invasion of resistant cells treated with inhibitors of: AKT (5 µM MK2206), EGFR (5 µM lapatinib), MET (1 µM foretinib), metalloproteinases (25 µM GM6001), and ABCA1 (12,5 µM probucol). The results' quantification is reported as the mean relative wound closure obtained from at least three independent tests ± SD. Statistical significance was defined as p ≤ 0.05 (*), p ≤ 0.001 (***), and p ≤ 0.0001 (****).

**Used methods:**

**Cell treatment with selected inhibitors**

Resistant melanoma cells were treated for 48h with 0,4µM vemurafenib, 0,4µM cobimetinib, and additionally one of the following inhibitors: 1µM foretinib (MET inhibitor), 5µM lapatinib (EGFR inhibitor), 5 µM MK2206 (AKT inhibitor), 25 µM GM6001 (MMPs inhibitor), or 12,5 µM probucol (ABCA1 inhibitor). Then, cells viability was assessed using the XTT test (described below), while their migration and invasion abilities were examined based on 2D and 3D wound healing assays (described in the manuscript).

**XTT assay**

A Cell Proliferation Kit II (XTT (2,3-bis-(2-methoxy- 4-nitro-5-sulfophenyl)-2 H-tetrazolium-5-carboxanilide); Roche) was utilized to measure the viability of cells treated with the drugs and the proliferation of cells after vemurafenib and cobimetinib withdrawal. To determine the proliferation rate, XTT was added to all of the investigated samples at time 0 (T0), and after 48h (T48) of cells’ growth. XTT mixture was added to the cells, and next they were incubated for 3h at 37 °C in 5%CO2/95% humidified air. Next, the absorbance at 450 nm was measured by a μQuant microplate spectrophotometer (Bio-Tek Instruments, Inc.) using Gen5 software (ver. 2.05, Bio-Tek Instruments, Inc.). The obtained values were then background corrected. The proliferation rate of the tested cells was calculated by dividing T48 by T0. Control cells’ proliferation was assumed to be 1. Each condition was performed in triplicate, and all experiments were conducted at least three times.

To evaluate the viability of cells treated with the drugs 48 h after inhibitors’ addition, the cell media were replaced with fresh ones, and the XTT mixture was added. Next, cells were incubated for 3h, and the absorbance was then measured as described above.
